# Evolution of Tandem Repeat Satellite Sequences in Two Closely Related *Caenorhabditis* Species. Diminution of Satellites in Hermaphrodites

**DOI:** 10.3390/genes8120351

**Published:** 2017-11-28

**Authors:** Juan A. Subirana, Xavier Messeguer

**Affiliations:** 1 Department of Computer Science, Universitat Politècnica de Catalunya, Jordi Girona 1-3, 08034 Barcelona, Spain; peypoch@cs.upc.edu; 2 Evolutionary Genomics Group, Research Program on Biomedical Informatics (GRIB)—Hospital del Mar Research Institute (IMIM), Universitat Pompeu Fabra (UPF), Doctor Aiguader 86, 08003 Barcelona, Spain

**Keywords:** satellite DNA, tandem repeat sequences, *Caenorhabditis nigoni*, *Caenorhabditis briggssae*, hermaphroditism, nematodes

## Abstract

The availability of the genome sequence of the unisexual (male-female) *Caenorhabditis nigoni* offers an opportunity to compare its non-coding features with the related hermaphroditic species *Caenorhabditis briggsae*; to understand the evolutionary dynamics of their tandem repeat sequences (satellites), as a result of evolution from the unisexual ancestor. We take advantage of the previously developed SATFIND program to build satellite families defined by a consensus sequence. The relative number of satellites (satellites/Mb) in *C. nigoni* is 24.6% larger than in *C. briggsae*. Some satellites in *C. nigoni* have developed from a proto-repeat present in the ancestor species and are conserved as an isolated sequence in *C. briggsae*. We also identify unique satellites which occur only once and joint satellite families with a related sequence in both species. Some of these families are only found in *C. nigoni*, which indicates a recent appearance; they contain conserved adjacent 5′ and 3′ regions, which may favor transposition. Our results show that the number, length and turnover of satellites are restricted in the hermaphrodite *C. briggsae* when compared with the unisexual *C. nigoni*. We hypothesize that this results from differences in unequal recombination during meiotic chromosome pairing, which limits satellite turnover in hermaphrodites.

## 1. Introduction

Hermaphroditism is widespread in different biological groups, involving an amazing number of strategies. Its study “challenges our understanding of sex determination and the specification of male and female reproductive function” [1]. In particular, a transition to hermaphroditism has appeared at least three times within *Caenorhabditis,* with XX hermaphrodites and XO males [2]. The sex-determination pathway is changed in a subtle manner upon the evolution from a unisexual (male-female) to a hermaphrodite species, as recently reviewed by Ellis [3]. The changes which may appear in the non-coding part of the genome are a particular aspect of this evolution, which we will analyze in this paper.

Satellites are tandem repeat sequences which are long (>100 nucleotides (nt) approximately) and have long repeats (>10 nt). Their role in chromosomal organization and evolution in different species has been recently reviewed by Garrido-Ramos [4]. Nematodes are a special case, since they contain a large number of different satellites [5]. Their study provides a handle to determine evolutionary changes in non-coding regions of the genome. Recently the whole genome sequence of the unisexual *Caenorhabditis nigoni* has become available [6]. It provides the opportunity to compare it with the closely related *Caenorhabditis briggsae* hermaphrodite [2], which has a smaller genome and determines the difference in non-coding regions in both species. Previously we reported on the large differences in the distribution and sizes of satellites in several *Caenorhabditis* species, which indicated a high evolutionary turnover, in particular of satellites with long repeat lengths [7]. No relation was found between the sequences of most satellites in different species, probably because they were distantly related. The availability of the new *C. nigoni* genome provides a unique opportunity to study the evolution of satellites in two closely related species. 

**Figure 1:**
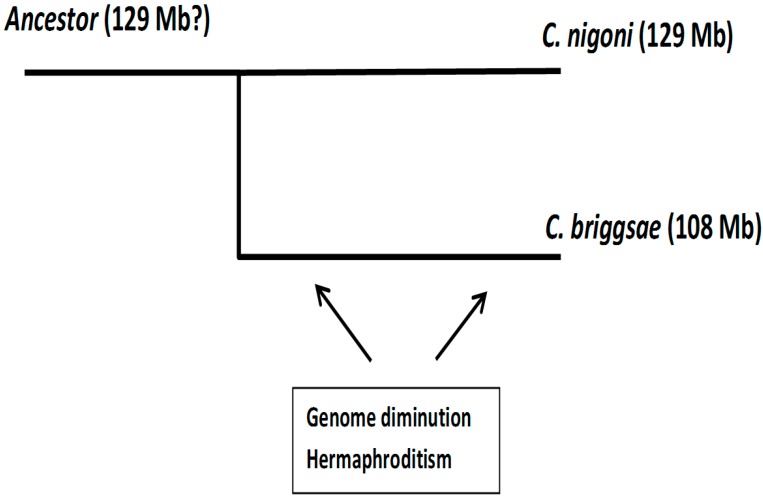
Phylogenetic relationship of *Caenorhabditis briggsae* and *Caenorhabditis nigoni.*

The scheme presented in Figure 1 sets a framework for our study. It indicates that both species diverged about 3.5 million years ago from a unisexual ancestor species [8]. Crosses between both species have been used to understand the genetics of reproduction isolation [9]. Since hermaphroditism only involves a few changes in the genetic control of gonad differentiation [10,11], the most logical hypothesis is that hermaphroditism appeared first and genome diminution occurred throughout the evolution of the *C. briggsae* species. Genome reduction involved a loss of protein-coding genes, mainly involved in reproduction [12,13,14,15]. Obviously, we do not know the size of the ancestor genome but based on a comparison between several *Caenorhabditis* species [14,15], we may assume that it had a size similar to other related unisexual species—thus, the size of the ancestor genome should be expected to be similar to *C. nigoni*.

Following our previously reported methodology [7], we have determined the sequence and size of all satellites in both species and classified them in different groups or families. We find families shared by both species, as well as families unique for each of them. The detailed study of some of these families allows us to draw a clear picture of their mode of expansion, transposition and elimination in two species which are evolutionarily closer: this will give us some clues on the mechanisms of evolution of satellites from the common ancestor.

An additional feature of nematode genomes is the presence of numerous interspersed repeats. They were identified as repeated sequences with more than ten copies in the genome, which are distributed in 500 families, with 159,216 occurrences in total. They occupy about 3 Mb in the *C. briggsae* genome [16]. Individual repeats differ in length and only conserve part of the reference sequence of its family. Their eventual role in the genome is not known, although some of them have a sequence related to known transposons. In general, each family has a unique sequence without internal repeats but a few of them are related to the satellites which we study in this paper.

Our results contribute to the annotation of poorly characterized non-coding regions of the genome and their changes upon the appearance of hermaphroditism. Our findings are consistent with subtle changes in the mechanism of recombination upon the appearance of hermaphroditism.

## 2. Materials and Methods

### 2.1. Genome Sequences and Alignments

We have used the WS247 version of the genome of *C. briggsae*, downloaded from Wormbase [17], which derives from the sequence published by Ross et al. [18]. It contains six scaffolds assigned to chromosomes, five scaffolds tentatively assigned to chromosomes and one scaffold consisting of sequences without a chromosomal assignment. These scaffolds contain many unknown regions, with 3,003,126 nt of indeterminate (N) sequence out of a total genome size of 108,419,665 nt. The *C. nigoni* genome has become available only recently [6]. It contains six chromosomal scaffolds and 149 additional unplaced scaffolds, with a total length of 129,488,540 nt. This genome assembly has far less undetermined sequence than that of the *C. briggsae* genome assembly, since it has only 53,153 nt of N residues. Also, the alignment methodology used for the *C. nigoni* genome assembly should provide a more accurate determination of tandem repeat lengths.

Chromosomes and chromosome fragments of the two species were aligned with the M-GCAT program [19,20]. The dot-plot program DOTTER was also used for local alignments [21,22].

### 2.2. Satellite Identification

Satellites have been detected with the SATFIND program, which is available in our website [23] and is described in great detail elsewhere [7,24]. This program allows a precise definition of satellites (repeat size, number of repeats and internal regularity), which is not easily obtained with other programs such as those used in Wormbase [17]. We have adjusted the parameters in order to capture short satellites with at least three repeats, whereas in previous studies we only studied longer satellites, with a minimum of ten repeats. Furthermore, we have only accepted those satellites that have ≥60% of their repeats with an identical length. In this way, most irregular satellites are eliminated, although with these parameters some satellites with only three repeats may still be irregular. Occasionally we have also changed the parameters of the program to detect additional satellites with a decreased regularity. We have further limited our study to repeats shorter than 300 bases, since there are very few satellites with longer repeats in the two species under study. A description of all satellites in different formats is provided (Supplementary Satellite Data Folder).

Each satellite has also been characterized by a similarity score obtained upon alignment of all its repeats which have an identical size, thus excluding all repeats with indels. Each satellite may be also characterized by a homogeneity parameter which gives the proportion of repeats with the same length in each satellite. This parameter varies between 0.6 (60%) and 1.0 (100%), since satellites with low homogeneity have not been accepted, as mentioned above. 

### 2.3. Identification of Satellite Families

In order to detect related satellites, we have used MALIG, a progressive multiple sequence alignment algorithm, which we have developed to align satellite repeats and identify families of satellites with a related sequence, available in our website [23,24]. It has been described in detail elsewhere [7]. The program considers reverse sequences as well, normalizes the alignment score to the maximum possible value and selects the cyclic permutation with the highest score. Then the progressive multi-alignment is applied to the matrix of pairwise alignment scores. The process finishes when the score is smaller than a similarity threshold (input parameter) which we set to 0.6.

We have searched for families taking satellites from both species together. Each family is characterized by three values; Fam_*a*_*b*_*c*. The order in the list of families is given by *a*, starting with those families with the largest number of members. The second value *b* gives the size of the repeat; *c* gives the number of members in the family. We have searched for families in three groups, depending on the size of the repeats: ≤27, 28–99 and ≥100 nt. The consensus sequence of the repeat in each family is calculated taking into account the circularly permuted sequence of all repeats. Individual families may contain satellites from either one or both species. We have mainly analyzed those families with repeats ≥28 nt. Details of the alignment process and consensus sequences are given in the Supplementary Satellite Data Folder.

### 2.4. Comparison of Syntenic Satellites 

Some of the satellites from an individual family may be found in syntenic positions in the two species. We created a data base of syntenic regions between *C. briggsae* and *C. nigoni* using M-GCAT [19] and obtained a complete genome alignment similar to the data reported in the original *C. nigoni* genome sequence [6], as shown in Supplementary Figure S1; it demonstrates a clear linear alignment between both genomes. When a satellite belongs to a syntenic region, we aligned it with the syntenic region in the other species and determined the expected position of a related satellite. In some cases, a clear correspondence between satellites of the two species was detected. In other cases, only a partial similarity was found, which may indicate the presence of an irregular or degraded satellite. The significance of such similarity was determined by comparing our alignment score (percentage of identities) against the score obtained in a random model in which the syntenic region is randomly selected. When the score is greater than the random model score, it indicates the presence of a partially degraded or imperfect related satellite. We carried out these alignments in both directions, either from *C. briggsae* to *C. nigoni* or vice versa and selected alignments with an *E*-value < 0.001. We used stringent parameters, so that the presence of a related satellite should not be missed but with this approach, low values of the alignment score (below 55%) should still be individually verified.

## 3. Results and Discussion

### 3.1. General Satellite Features 

We have detected 7803 satellites with at least three repeats in *C. nigoni* and 4834 in *C. briggsae*. A complete list is given in the Supplementary Satellite Data Folder. A list of the longest satellites is also given (Supplementary Tables S1 and S2). About 90% of satellites have short repeats (≤27 nt). The main difference between both species is the larger number and length of satellites in *C. nigoni*, practically for all repeat sizes (Table 1). However, it should be noted that the actual length of long satellites is not reliable, since all the programs used to determine the genome sequence have alignment problems for long repetitive sequences. 

**Figure 2:**
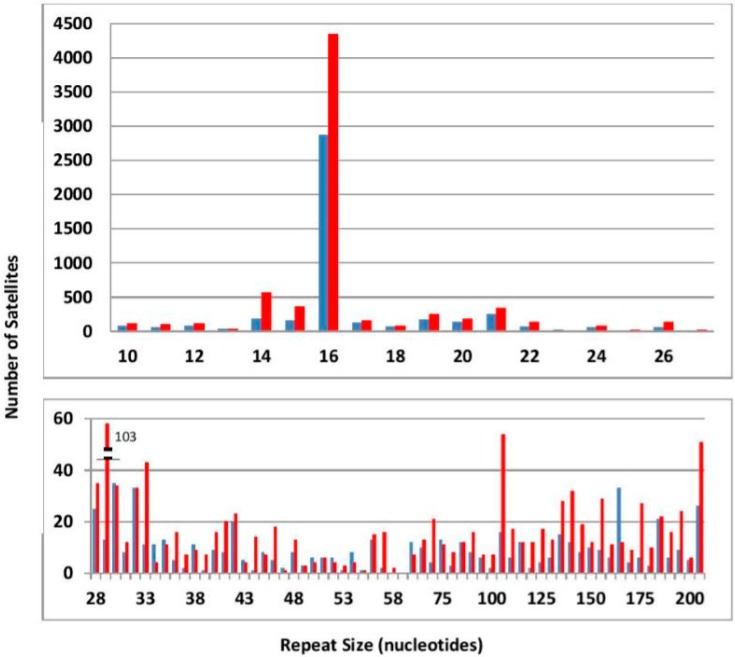
Distribution of the number of satellites as a function of their repeat size in nucleotides (nt). Values for *C. briggsae* are in blue; for *C. nigoni* in red. The data are presented in two separate frames for repeats either shorter or longer than 28 nt. For lengths above 60 nt the data have been merged in beans of 5 nt.

As mentioned above, there is a very clear excess of satellites in *C. nigoni* with respect to *C. briggsae*, even after normalization by genome size. The only remarkable exceptions are satellites with a repeat of 163 bases, which are much more abundant in *C. briggsae* than in *C. nigoni* (Figure 2). A family of satellites with this 163 repeat is found in the X chromosome, to be described below. Most long satellites show a higher order structure, as previously described in other species [25], including *Caenorhaditis. elegans* [7]. Such higher order structure is usually characterized by identical groups of a few repeats within a satellite. An example is given in Supplementary Figure S2. Satellites are not evenly distributed; they accumulate at both ends of each chromosome. This tendency is less pronounced in the X chromosome.

**Figure 3:**
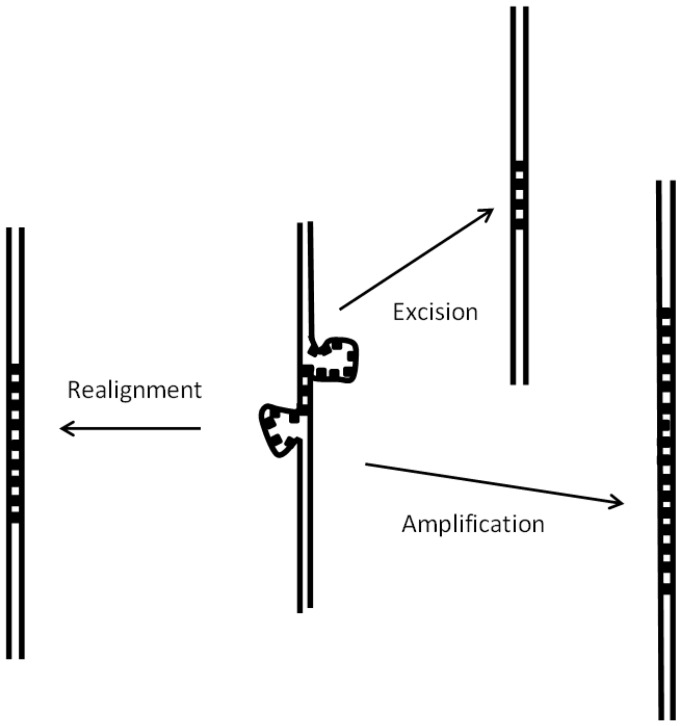
Mispairing of satellite repeats in meiosis. Satellite loops may appear when the repeats are not paired in register. The most frequent outcome of such situation will be a realignment to place in register the two satellites. Alternatively, the situation may be resolved in two other ways—either excision of the unpaired loops or amplification by repair synthesis. As a result, changes in the number of repeats of the satellites and even their disappearance will occur. The two latter cases will necessarily be found when the two homologues have a different number of repeats, a situation which will occur frequently in unisexual species.

Each satellite has been characterized by the variability of sequence of its repeats, measured by its similarity score, which is also given in Tables S1 and S2. This variability is determined by two antagonic effects: growth by unequal recombination and accumulation of mutations. Unequal recombination, illustrated in Figure 3, stabilizes the repeat sequence, which results in a high similarity score in long satellites; practically all satellites longer than 2 Kb have a similarity score greater than 0.8. In the case of short satellites the similarity score is variable. On one hand, they may have a low score due to accumulated mutations, which may prevent their further growth by unequal recombination and favor their degradation. On the other hand, short satellites with a high similarity score correspond to satellites which have appeared recently and have not accumulated mutations.

As a particular case, we searched for telomere repeats (TTAGGC/GCCTAA) [26] and found that they are abundant throughout the genome of both species but only occur in two cases at the ends of chromosomes: the start of chromosome II in *C. nigoni* and the end of chromosome III in *C. briggsae*. This result is probably due to difficulty in sequencing the terminal regions of chromosomes. Alternatively, telomere sequences may deviate from the consensus sequence found in other nematodes.

For reasons mentioned above, we have not studied in detail satellites with short repeats. However, we should mention the very abundant repeat of 16 nt (Figure 2). Most satellites with this repeat correspond to an approximate octamer dimer of the sequence ARATTCWG. This sequence does not appear randomly but in clusters (Supplementary Table S3). This uniform distribution of sequences is an indication of frequent recombination events. Similar satellites are absent in other *Caenorhabditis* species [7].

### 3.2. Satellite Families

Next, we identified families of satellites with related repeat sequences, considering both species together. We assume that the satellites in each family have a common origin. We obtained a total of 194 families which contained at least two related satellites with a repeat length ≥28 nt (Supplementary Table S4). Satellites with repeats of these sizes can be compared more easily between species [7] and are the main focus of this study. We also found 573 unique satellites (362 in *C. nigoni* and 211 in *C. briggsae*), which have no related satellite in either of the two species (Supplementary Satellite Data Folder). The distribution of satellites in the different families is very uneven. In particular, several of the largest families predominate in one of the two species, with no or few members in the other species. In cases where a similar number of satellites from one family are found in both species, the satellites are usually not found in syntenic regions. A study of those families which predominate in one species is of particular interest to understand the mechanisms of transposition and disappearance of satellites; we will present some cases below. Satellites in the same family are usually distributed over different chromosomes but many of them are clustered in neighboring positions. This suggests that there are two mechanisms of transposition, one between neighboring regions and another one between distant regions of the genome. We should note here that transposition of coding sequences in *Caenorhabditis* usually takes place within the same chromosome [27], whereas transposition of satellites follows a different pattern, since it frequently involves different chromosomes. This behavior differs from what is found in eutherian species, where exchange of chromosome fragments is frequent [28].

The existence of satellite families suggests an alternative way to divide satellites into two groups: unique satellites and transposable satellites. When a satellite appears, it may remain in its original position as a unique satellite. Alternatively, it may be transposed to other regions of the genome and form a family of satellites with a related sequence. By this token we find that unique satellites predominate over transposable satellites in both species (77.2% in *C. nigoni* and 77.7% in *C. briggsae*). We conclude that once a satellite is formed, its probability of being transposed is only 22%–23% in both cases. However, as a result of transposition, individual satellites that belong to transposable satellite families predominate over those that belong to unique satellite locations, again in the genomes of both species; 60% of all individual *C. nigoni* satellites are in transposable satellite families, as are 53% of all individual *C. briggsae* satellites.

**Figure 4:**
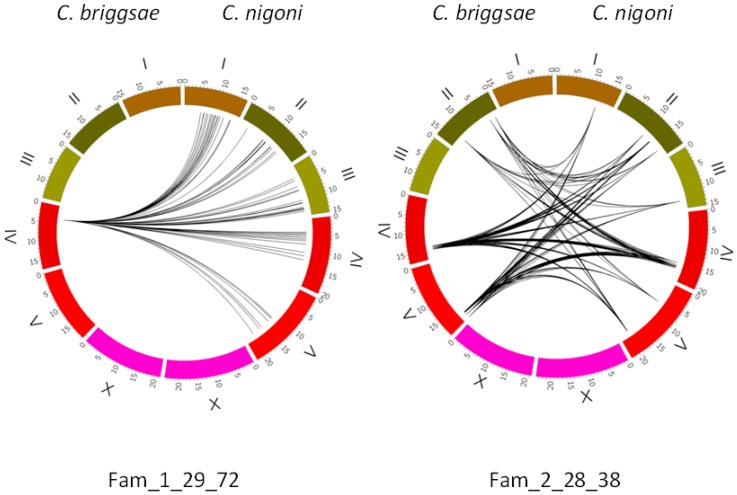
Comparison of two families of satellites with repeat length 28–29 nt. Genome coordinates are given in mega-bases for each individual chromosome. The 1_29_72 family has a single satellite in *C. briggsae*, with a related satellite in a syntenic position in *C. nigoni* at IV: 4916893. The 2_28_38 family has its satellites spread over both genomes; some of them show a clear synteny between both species (Supplementary Table S5), whereas in other cases a partial synteny, with imperfect-degraded satellites is also found (Supplementary Tables S6 and S7). The curved lines in the central part of the figure bind the satellites of the same family in both species.

As an example of the variability of individual satellite families, we present here two families which are clear examples of different evolutionary stories; both are absent in the X chromosome. The position of their satellites is given in Figure 4. Some clusters of satellites are clearly apparent. Fam_1_29_72 has evolved only recently in *C. nigoni*, as shown by the conservation of its 5′ and 3′ adjacent regions in most of its members (to be discussed below). A single satellite in this family is found in *C. briggsae*; it presents a clear synteny with one satellite in *C. nigoni*. A likely explanation is that a single satellite was present in the ancestor species and was conserved in *C. briggsae*, whereas in *C. nigoni* it was actively transposed and a whole family of new satellites appeared. Fam_2_28_38 is a rare case in which a large family present in the ancestor has been preserved and has evolved in both species. Some of its original members have been conserved in syntenic positions, while other satellites from the same family have appeared in different positions.

In summary, the overall comparison of the two species which we have presented clearly shows that *C. nigoni* has more and longer satellites (Table 1, Figure 2 and Supplementary Tables S1 and S2). Even more significant is the difference in the number of members in satellite families (Supplementary Table S4). Exclusive families which have their satellites only in one species have probably grown recently; we find 29 exclusive families in *C. nigoni* and only 7 in *C. briggsae*, by comparing all families with more than two members. In conclusion, all these features indicate that a significant decrease in the number and turnover of satellites has occurred in the course of differentiation of *C. briggsae* from its unisexual ancestor.

### 3.3. Conserved Pairs of Satellites

Satellite pairs which are found in syntenic regions of the two species and have related repeat motifs are likely to have a common origin. The differences between such satellite pairs provide a measure of the rates of mutation and recombination during the time elapsed since the two species diverged from their common ancestor. Changes in the length of satellites are not so informative about this process, given the very high frequency at which unequal recombination occurs and changes satellite length. The alignment of contigs used to determine the genome sequence may also result in incorrect lengths of long satellites, as mentioned above (Section 3.1).

A detailed comparison of several evolutionary related satellite pairs is given in Supplementary Table S5. Some of them are unique, in the sense that there is a single satellite in each species. In other cases, conserved satellite pairs belong to a satellite family with several members in both species but in general only one member is found in a syntenic position. Only two families (2_28_38 and 3_30_21) have several conserved pairs in both species. All these related satellites have similar mutation and recombination rates in the two species (Supplementary Table S5), which demonstrate a continuous evolution from the ancestor species.

Hypothetically, some of the syntenic satellite pairs may correspond to conserved proteins, in particular if the repeat size is a multiple of three and identity is high. However, we have only detected one clear such case, with a repeat size of 285 bases, which corresponds to a fragment of an ortholog of the *C. elegans* tag-80 gene. The two satellites with a repeat size of 102 bases have a high internal similarity (Supplementary Table S5) and might correspond to a protein with an internal repeat of 34 amino acids but we have not detected any clear sequence relationship with the tetratricopeptide reference sequence [29] in any of the six possible reading frames of the satellite repeat.

Another aspect of synteny is due to the fact that some satellites present in the ancestor species may disappear by accumulation of mutations which disrupt their repetitive sequence only in one of the two species under study; remains of the satellite may be found in the other species. We have investigated this possibility by determining if there is any relation between the sequence of the satellites in one species and the sequence of its syntenic region in the sister species (Supplementary Tables S6 and S7). We find remains of satellites in some cases, which indicates that in both species the absence of some satellites is due to their internal degradation.

### 3.4. Birth and Growth of New Satellites

New satellites are formed in a progressive manner in germ cells. The first step in the birth of a satellite should be one duplication event, so in principle any sequence may become a satellite. However, it is likely that satellite expansion is favored by local sequence features, such as short A-tracts, hairpins or palindromes [30,31,32], which are frequently found in many satellites. Microsatellites, which are frequently found near satellites (Table 2, Supplementary Figure S3), may also facilitate satellite growth [33]. Further growth may be due to replication slippage, as it has been suggested for microsatellites [34]. Repeat expansion may also take place associated with either DNA duplication or double-stranded break repair, which may occur in a variety of forms [35,36,37,38]. This possibility has been studied in some *C. elegans* mutants [39]. 

An additional mechanism for satellite growth is unequal recombination. It usually takes place with segments of 100–300 bases in length [40,41], which correspond to a few satellite repeats. Anomalies in recombination in *C. elegans* have been reviewed by Zetka [42]. An alternative mechanism is expansion of satellites through Okazaki fragments, which usually have an approximate size of 200 bases and may be duplicated during DNA synthesis. This possibility has been investigated in yeast by Shah et al. [43]. In the case of very long satellites, duplication of a long stretch containing many repeats may take place in a way similar to gene duplication, which is very common in *C. elegans* [44]. Several additional processes may be involved in the duplication of genome fragments, such as DNA transposition and retro-transposition [45,46].

As mentioned above, both genomes contain satellites which are unrelated with any other satellite and occur only once (Supplementary Satellite Data Folder). The number of such unique satellites is significantly larger in *C. nigoni*, as mentioned above. The difference between both species is much more significant if we compare satellites greater than 1 Kb, with repeat lengths ≥28. It turns out that there are 43 satellites of this type in *C. nigoni* but only 5 in *C. briggsae*. Inspection of Supplementary Tables S6 and S7 shows that most of these unique satellites have no related syntenic region in the other species. This observation indicates that *C. nigoni* has a capability of producing new longer satellites more efficiently than *C. briggsae*. The alternative explanation that these satellites were already present in the ancestor species and disappeared in *C. briggsae* is unlikely, since all these satellites display a high internal similarity, which indicates a recent formation (Supplementary Satellite Data Folder).

### 3.5. New Satellite Families: Precise Tandem Repeats and Conserved Adjacent Sequences

Satellite families which only appear in one of the two species have probably arisen after the divergence of the two lineages; they may be considered young families. Their recent appearance may indicate a burst of transposition. Transposition of satellites may involve adjacent regions with a transposon activity. With this idea in mind we have investigated whether satellite families have conserved adjacent regions in either the 5′ or the 3′ end of individual satellites of the same family but we found no cases in *C. briggsae*. However, we found seven families which had conserved 5′ or 3′ regions in *C. nigoni*, (Table 2). Only in two of these families a single related satellite was found in *C. briggsae*. We find that most of them have a very regular structure, with an exact number of repeats and identical 5′ and 3′ adjacent sequences which span about 500 bases. These features indicate that these satellite families are young: they have developed recently and have not undergone a significant number of changes. In Table 2 one can clearly appreciate that the conserved adjacent regions differ for each satellite family. This fact indicates that transposition does not require identical adjacent regions. We next describe in detail one of these families.

The 4_137_15 family contains 15 members in the genome of *C. nigoni* and no member in *C. briggsae*. They are distributed over several chromosomes. A characteristic feature of the satellites in this family is that all of them contain almost identical satellite repeats, as judged by their high similarity score (average value 0.976). Furthermore, each of them starts with the same seed sequence GATGTTTGTG, which indicates that there is no circular permutation of sequence in different satellites; they also have an exact number of repeats (no partial repeats). These features indicate a recent appearance; they have not undergone mutations which disrupt their regularity. 

The similarity between satellites covers also the adjacent 5′ and 3′ genome regions, which may extend up to 500 bases (Supplementary Figure S3). All these features are independent of the number of repeats in the satellites, which varies between 3 and 16 repeats. An exception are the two members of this family found in the X chromosome, which are longer (33 and 37 repeats) and have different adjacent regions, although the sequence of their repeats is practically identical to the repeats in the rest of the family.

We tried to determine the corresponding region in the genome of *C. briggsae* and found that in most cases the whole region around the *C. nigoni* satellite was not present. Apparently, most of the satellites in this family were either not present or are found in regions of the genome which have been lost in *C. briggsae*, with five notable exceptions, given in Table 3. 

**Figure 5:**
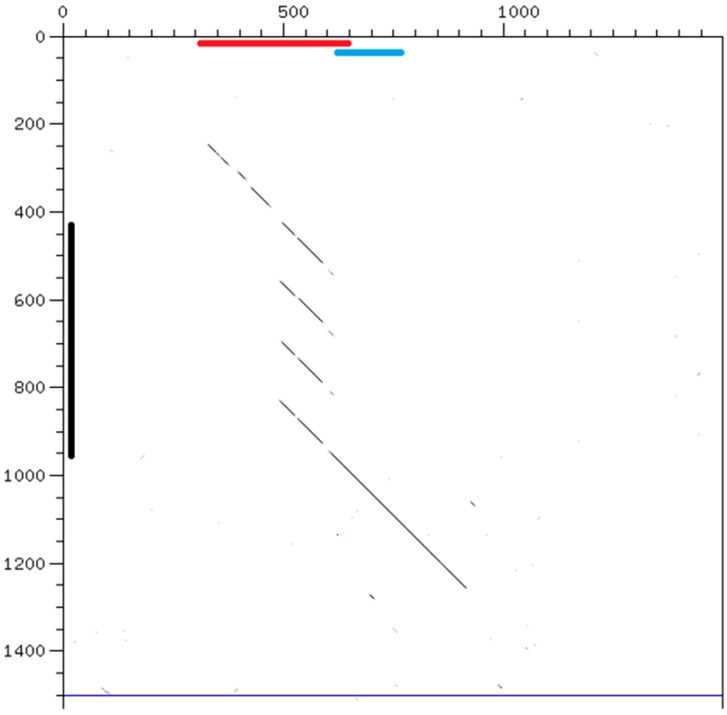
Dot-plot alignment of 1500 nt of corresponding regions of chromosome V: horizontal, *C. briggsae*, starting at position 19,137,001; vertical, *C. nigoni*, starting at position 21,406,001. The black line indicates the position of one satellite of *C. nigoni* with a 137 repeat (Cnigo_V:21406428). The red line indicates an interspersed 468 sequence and the blue line a 196 sequence, placed at the positions given in Wormbase [17]. The *C. nigoni* satellite appears in a syntenic region where a related 468 sequence is found in *C. briggsae*.

Interestingly, in the satellites of this family which have a corresponding region in *C. briggsae*, it contained matching sequences with 468 and 196 interspersed repeats. The sequence and position of these repeats can be recovered from Wormbase [17]. We should note that they are abundant in the genome of *C. briggsae* [17]: 53 occurrences for 468 and 348 for 196. Their average length is close to 200 nt in both cases but individual occurrences vary between 35 and 900 nt. It turns out that the 468 sequences show a strong synteny with the 5′ adjacent region plus one satellite repeat in *C. nigoni*, whereas the 196 sequence corresponds to the 3′ adjacent satellite region. An example is given in Figure 5. We have named proto-repeat the consensus of the 468 sequences which shows a match with the *C. nigoni* satellite repeat; it is clear that there is a strong correspondence but with significant differences (Supplementary Figure S4). A related satellite family 33_107_4 is also described in Supplementary Figure S4.

In view of these results we analyzed in detail the longest 468 regions in *C. briggsae* in order to determine whether any of them contained related satellites. We only found a few satellites with a similar repeat that had not been detected with the SATFIND program because they either had only two repeats (IV_16681956 and X_20322118) or had an irregular sequence (X_1819875). 

**Figure 6:**
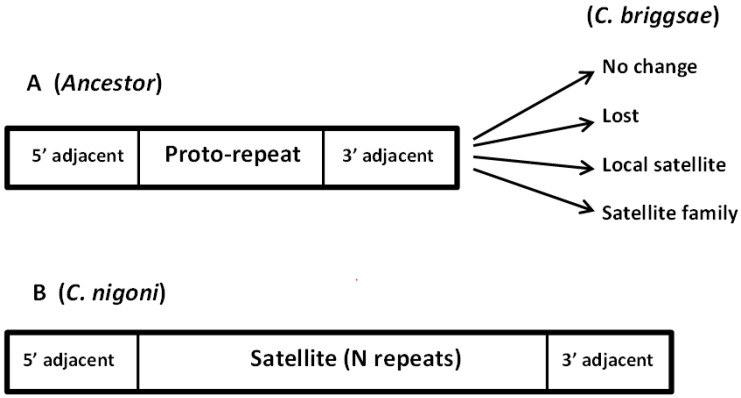
Evolution of young satellite families. As an example, we present the case of Fam_4_137_15, discussed in detail in the text. This family is only found in *C. nigoni* and presents conserved 5′ and 3′ adjacent sequences (B). In the syntenic position in the ancestor genome (A), a single proto-repeat was found between the conserved adjacent regions. Such regions evolved in several ways in *C. briggsae*, as indicated by the arrows. Only in *C. nigoni* a satellite family appeared, with a sequence related to the proto-repeat sequence. In some of the satellite positions, a syntenic region is found in *C. briggsae* with a single proto-repeat. An example is presented in Figure 5. The proto-repeat plus the 5′ adjacent sequence correspond to the 468 sequence; the 3′ adjacent sequence corresponds to the 196 sequence.

In summary, in all cases where we find a correspondence of sequence of the *C. nigoni* satellites from the 2_137_16 family, we have detected a single proto-repeat in *C. briggsae*, together with related 5′ and 3′ adjacent sequences. A scheme of the organization of these satellites is presented in Figure 6. We may now ask which was the most likely situation in the ancestral species?

The high identity of sequence of these satellites indicates that they have emerged only recently in *C. nigoni*, since they have not accumulated mutations. Also, their uniformity and the significant difference in sequence from the proto-repeat, indicates that they have not grown independently at their positions. We tentatively conclude that in the ancestor there were already 468–196 interspersed repeats, some of which have been conserved in both species. In *C. nigoni* a single satellite appeared in one of these sites with a related different sequence; it was later transposed to different sites in the genome. In particular it appears that the 468–196 sequence is a mark which signals a site for a favorable transposition of these satellites.

We have also detected another family (Fam_11_155_10), only found in *C. nigoni*, which has apparently grown from a proto-repeat in the ancestral species, which is only conserved in one position in *C. briggsae* (Supplementary Figure S5).

### 3.6. The X Chromosome Contains Characteristic Satellites 

**Figure 7:**
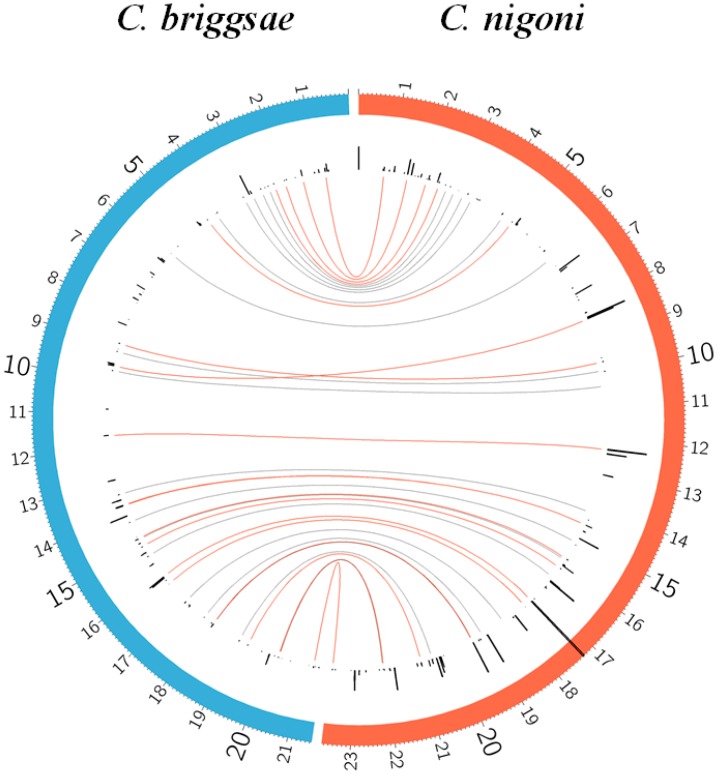
Distribution of satellites in the X chromosome. All satellites in the X chromosome of both species are represented as black lines proportional to the satellite length. Genome coordinates are given in mega-bases. The longer average size of satellites in *C. nigoni* can be easily appreciated. The curves in the central region indicate the correspondence of satellites. Lines in red indicate conserved satellites (Supplementary Table S5). Lines in grey indicate the correspondence of a *C. briggsae* satellite with a region in *C. nigoni* with a significant sequence identity, corresponding to a partially degraded or irregular satellite (Supplementary Table S6). The longest satellite in *C. nigoni*, at position 17271358, has a length of 31.5 Kb. Short satellites (<500 nt) are visible by applying a significant zoom to the figure.

A comparison of the satellites in the X chromosome is presented in Figure 7. This chromosome, as expected, has a higher level of synteny than the autosomes (Supplementary Table S5). A characteristic feature of its satellites is the presence of several families only found in this chromosome. In fact, only 9% of the satellites with repeats ≥28 in the *C. briggsae* X chromosome belong to families shared with the autosomes. It appears that there is a limited exchange of satellites with the autosomes, since there are also many families in the autosomes, which are absent in the X chromosome; examples are given in Figure 4.

Fam_3_30_21 and 3_163_19 are the predominant families in the X chromosome and are not present in the autosomes. Fam_3_30_21 is shared with *C. nigoni*, whereas only one member of Fam_3_163_19 is found in *C. nigoni* in a syntenic position but with a large difference in size (Supplementary Table S5). Another family, 5_182_14, is also restricted to the X chromosome and is also shared with *C. nigoni* but no synteny has been detected, although half of its satellites are found in unplaced contigs.

The 3_163_19 family has a relatively large number of satellite members but eight of them are found in the chromosomally unassigned scaffold 8, with a total of 29,693 bases; we suspect that some of the satellites found in scaffold 8 may actually correspond to satellites in the X chromosome. Its repeat of 163 bases appears in Wormbase [17] as SAT1-CB. Synteny has only been found in one satellite of this family, which does not appear to have been transposed in *C. nigoni* and possibly derives from a single satellite in the ancestor species; no synteny is present in the other satellites in this family (Supplementary Table S5). This may be a unique case of transposition activity in a *C. briggsae* satellite family, although its satellites have no conserved adjacent 5′ and 3′ sequences.

### 3.7. Satellites and Hermaphroditism

Hermaphroditism has been associated with gene loss, including many genes involved in reproductive function [12,13,14]. Here we have found an increased loss of satellites in *C. briggsae* when compared to the related unisexual species *C. nigoni*. Below we discuss possible factors influencing this pattern, other than general genome shrinkage.

The first step in satellite growth is the duplication of a short sequence due to defects in DNA synthesis, as discussed in detail in Section 3.4. The second step is further growth by unequal recombination. Differences in these processes in hermaphrodites may result in a decrease of satellite growth, when compared with unisexual species, as we have reported here. As shown in Figure 3, mispairing of a satellite may be repaired in different ways. In the case of unisexual species, homologous satellites of different length may pair in meiosis, so that repair must take place by either amplification or excision; as a result, satellites of different lengths will appear in the progeny. In hermaphrodites such as *C. briggsae*, pairing in meiosis will usually take place between satellites with an identical length, so that in general repair will take place by realignment (Figure 3), without any change in satellite length. A further process which explains the larger number of satellites in *C. nigoni* is the recently reported increased number of double-stranded breaks during meiosis in unisexual species [47]: their repair may favor the appearance and growth of satellites.

Additionally, new satellite families are found more frequently in *C. nigoni* (Table 2). It appears that transposition remains more active in *C. nigoni* than in *C. briggsae*. We should also note that transposition of satellites between different chromosomes is common, since satellites of the same family are often dispersed throughout several chromosomes, as we have already described in *C. elegans* [5]. In contrast, orthologous genes are always found in the same chromosome [27]. This observation suggests that transposition of satellites involves unique mechanisms.

## 4. Conclusions

In summary, our results show that the number, length and turnover of satellites are restricted in the hermaphrodite *C. briggsae* when compared with the related unisexual *C. nigoni*, based on the following observations:

1. We have detected 4834 satellites in *C. briggsae* and 7803 in *C. nigoni*. Satellites with repeats ≥28 nt form 194 families of related satellites from both species. We also find 573 unique satellites, unrelated in sequence with any other satellite (362 in *C. nigoni* and 211 in *C. briggsae*).
2. Some satellites in *C. nigoni* have developed from a proto-repeat present in the ancestor species and conserved as an isolated sequence in *C. briggsae*.
3. The rate of neutral mutation in *C. briggsae* and *C. nigoni* since they diverged from their ancestor is practically identical, as judged by the changes found in conserved satellites (Supplementary Table S5).
4. Transposition of satellites creates satellite families. It follows a different mechanism than transposition of coding sequences, since satellites may be transposed between different chromosomes. An additional mechanism favors transposition of satellites between closely spaced genome regions, which gives rise to clusters of related satellites.
5. Transposition of satellites is limited in *C. briggsae*; very few new families are found when compared with *C. nigoni* (Figure 3).
6. Transposition of satellites to form new satellite families in *C. nigoni* is associated with conserved 5′ and 3′ adjacent regions at both ends of the satellites (Table 2).
7. The identical size of satellites during homologue pairing in meiosis in hermaphrodites may prevent its growth by unequal recombination (Figure 3).
8. Birth of new satellites is facilitated in unisexual species, with respect to hermaphrodites, since double-stranded break repair appears to be a major mechanism for the initial duplication of short nucleotide sequences.

## Figures and Tables

**Table 1:** Distribution of satellites in chromosomes.

Chromosome			Satellite Distribution			
Name	Species	Size (Mb)	Repeat 10–27	Repeat ≥28	Total	Satellites/Mb
I	*C. briggsae*	15.46	634	66	700	45.3
	*C. nigoni*	16.74	923	145	1068	63.8
II	*C. briggsae*	16.63	1172	63	1235	74.3
	*C. nigoni*	19.23	1904	145	2049	106.6
III	*C. briggsae*	14.58	500	67	567	38.9
	*C. nigoni*	15.54	740	129	869	55.9
IV	*C. briggsae*	17.49	497	71	568	32.5
	*C. nigoni*	20.39	816	134	950	46.6
V	*C. briggsae*	19.50	866	85	951	48.8
	*C. nigoni*	22.29	1360	141	1501	67.3
X	*C. briggsae*	21.54	279	101	380	17.6
	*C. nigoni*	23.65	482	125	607	25.7
Unplaced	*C. briggsae*	3.12	373	60	433	138.8
	*C. nigoni*	11.66	604	181	785	67.3
Total	*C. briggsae*	108.42	4321	513	4834	44.6
	*C. nigoni*	129.49	6829	974	7803	60.3

Note that the relative number of satellites in *C. nigoni* (60.3 Sats/Mb) is significantly larger than in *C. briggsae* (44.6 Sats/Mb). A correction for satellites eventually present in unplaced regions (Ns) in *C. briggsae* increases this number to 48.4 Sats/Mb, so that the relative number of satellites in *C. nigoni* is 24.6% greater than in *C. briggsae*.

**Table 2:** Conserved 5′ and 3′ adjacent regions in young satellite families.

Family	5′ Adjacent Region	Start-Satellite-End	3′ Adjacent Region
4_137_15	AATCGGTAGGACACAGTTCGAT	GATGTTTGTG---GACACCGTTC	ATGGGTTTTTGAAGGTGATCCC
33_107_4	CCCCAtTGTtCTCagTCTGATC	AGTATTCGCA---GCCTACTTTC	ACAAACGTCGAACAACTGAAaT
10_176_10	ATCAnCATCTTGAAAAAATCAA	AATTTTGACG---ATTTTTAGCG	variable
11_155_10	ATCTcCCctTTCCTCCTncCnC	TAGCATTCGA---CTCCTAACTC	variable
12_154_9	Variable	GTCCGTCCGA---ACTCTCTTTT	(TC)_n_CATCCACAATAGTCWGC
5_85_13	ACCTGACAAAAAACCCCTATCA	GGATATATGG---TCTRGCATCC	naGGGCGTCTnGCGACACCCTG

Only the consensus of the first 22 bases in the adjacent regions is shown, although a partial conservation of sequence extends for about 500 bases. A detailed presentation for Fam_4_137_15 is also available (Supplementary Figure S3). The satellites from Fam_12_154_9 present a microsatellite of variable length in its 3′ Adjacent region. Fam_1_29_72 is not included in the table; it also presents partially conserved 5′ and 3′ regions. Lower case is used to indicate that there are changes in this position, but the indicated base predominates.

**Table 3:** Correspondence of *C. nigoni* satellites, repeat 137, with 468 sequences in the genome of *C. briggsae*.

*C. briggsae*			*C. nigoni*
Chromosome	468 Sequence		Satellite Position
	Start	Size	
I	1610666	144	I_2234586
III	14143016	342	III_15035724
IV	241446	224	IV_317549
IV	17352776	169	IV_20134955
V	19137314	332	V_21406428

The positions of 468 sequences in the genome of *C. briggsae* are indicated. In all cases there is also a neighboring 196 sequence. The other satellites in the 4_137_15 family (11 satellites) are found in regions of the *C. nigoni* genome which have been either lost or not sequenced in the *C. briggsae* genome. The 468 sequence in chromosome I has additional 468 sequences within 1 Kb.

## Supplementary Materials

The following are available online at www.mdpi.com/2073-4425/8/12/351/s1 and www.mdpi.com/2073-4425/8/12/351/s2. Figure S1: Syntenic regions in each chromosome of *C. briggsae* and *C. nigoni*. Figure S2: Example of evolution of a satellite by mutation and growth by unequal recombination. Figure S3: 5′ and 3′ adjacent sequences in *C. nigoni* satellites of the 4_137_15 family. Figure S4: Comparison of the consensus sequence of the *C. briggsae* proto-repeat and the corresponding satellites in *C. nigoni*. Figure S5: Dot-plot of a satellite in *C. nigoni* compared with its syntenic region in *C. briggsae*. Table S1: Similarity score for *C. nigoni* satellites longer than 1 Kb. Table S2: Similarity score for *C. briggsae* satellites longer than 1 Kb. Table S3: Octamer repeats (ARATTCWG) in *C. briggsae* and *C. nigoni*. Table S4: All satellite families. Table S5: Comparison of conserved satellites. Table S6: Syntenic regions of *C. briggsae* satellites in the genome of *C. nigoni*. Table S7: Syntenic regions of *C nigoni* satellites in the genome of *C. briggsae*. Satellite data Folder: Description of all satellites and satellite families in several files with different formats.
