# Supplementary material for: Evolution of Tandem Repeat Satellite Sequences in Two Closely Related Caenorhabditis Species. Diminution of Satellites in Hermaphrodites
Source: Genes (Basel). 2017 Nov 28;8(12):351. doi: 10.3390/genes8120351 (PMC5748669; doi:10.3390/genes8120351)
Supplement: Supplementary file 1 [file genes-08-00351-s001.pdf]

Figure S1. Syntenic regions in each chromosome of *C. briggsae* and *C. nigoni*. Representation of syntenic regions in each chromosome of *C. briggsae* (blue) and *C. nigoni* (red). Regions of 500 syntenic base-pairs are joined by green lines. Overall synteny values are about 50 % for all autosomes and 60% for the X chromosome. The correspondence between both species is clearly lineal.

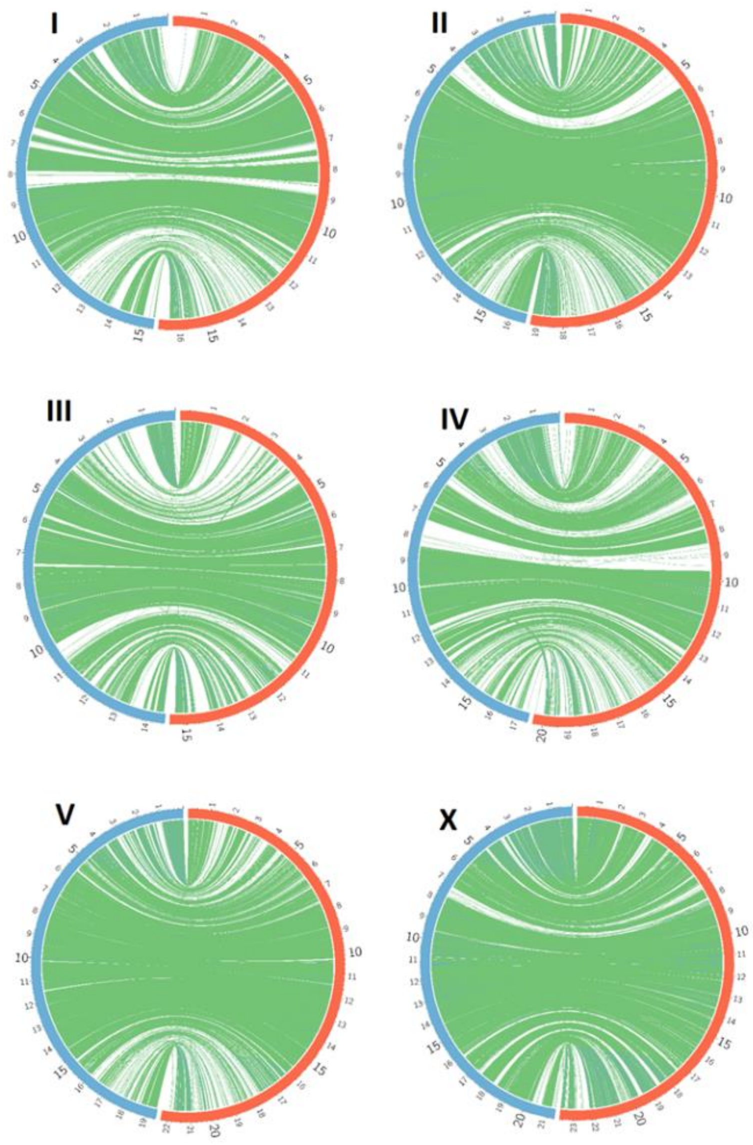

Figure S2. Example of evolution of a satellite by mutation and growth by unequal recombination. Colored regions have identical sequences. This satellite contains 21 different repeats which have appeared by recombination and subsequent mutations; they are numbered at the right side. Some of them (2, 5-8, 12) are found several times in the satellite. Recent recombination events involving several repeats are underlined.

>Cnigo.2016_X:22932995-22937528 Satlength=4534 Nr of Repeats=33 RepeatLength=137 seed=TAGTTGGTCT

TAGTTGGTCTAGTCGGTCATGCGGGCTACGGTTTGCTGGACGGCTTTATCACTAGACTGGGGCACATCCACGACTGCTGATAATCAAGCGGAGGACACCGTTCAATGTTTGTGAGGTGGGCCTGGTGGGCACTGGGC-1

TAGTTGGTCTAGTCGGTCATGCGGGCTACGGTTTGCTGGACGGCTTTATCACTAGACTGGGGCACATCCACGACTGCTGATGATCAAGCGAAGGGCACCGTTCGATGTTTGTGAAGTGGGCCTGGTGGGCACTGGGC-2

TAGTTGGTCTAGTCGGTCATGCGGGCTACGGTTTGCTGGACGGCTTTATCACTAGACTGGGGCACATCCACGACTGCTGATGATCAAGCGAAGGGCACCGTTCGATGTTTGTGAAGTGGGCCTGGTGGGCACTGGGC-2

TAGTTGGTCTAGTCGGTCATGCGGGCTACGGTTTGCTGGACGACGACATCACTAGACTGGGGCACATCCACGACTGCTGATGATCAAGCGGAAGACACCGTTCGATGTTTGTGAAGTGGGCCTGGTGGGCACTGGGC-3

TAGTTGGTCTAGTCGGTCATGCGGGCTACGGTTTGCTGGACGGCTTTATCACTAGACTGGGGCACATCCACGACTGCTGATGATCAAGCGAAGGACACCGTCCGATGTTTGTGAAGTGGGCCTGGTGGGCACTGGGC-4

TAGTTGGTCTAGTCGGTCATGCGGGCTACGGTTTGCTGGACGGCTTTATCACTAGACTGGGGCACATCCACGACTGCTGATGATCAAGCGAAGGGCACCGTTCGATGTTTGTGAAGTGGGCCTGGTGGGCACTGGGC-2

TAGTTGGTCTAGTCGGTCATGCGGGCTACGGTTTGCTGGACGGCTTTATCACTAGACTGGGGCACATCCACGACTGCTGATGATCAAGCGAAGGGCACCGTTCGATGTTTGTGAAGTGAGCCTGGTGGGCACTGGGC-5

TAGTTGGTCTAGTCGGTCATGCGGGCTACGGTTTGCTGGAAGACGACATCACTAGACTGGGGCACATCCACGACTGCTGATGATCAAGCGGAGGACACCGTTCAATGTTTGTGAGGTAGGCCTGGTGGGCACTGGGC-6

TAGTTGGTCTAGTCGGTCATGCGGGCTACGTGTTTGCTGGACGGCTTTATCACTAGACTGGGGCACATCCACGACTGCTGATAATCAAGCGGAGGACACTGTTCAATGTTTGTGAGGTGGGCCTGGTGGGCACTGGGC-7

TAGTTGGTCTAGTCGATCATGCGGGCTACGTGTTTGCTGGACGGCTTTATCACTAGACTGGGGCACATCCACGACTGCTGATGATCAAGCGAAGGGCACCGTTCGATGTTTGTGAAGTGGGCCTGGTGGGCACTGGGC-8

TAGTTGGTCTCGGGCTACGTGTTTGCTGGACGGCTTTATCACTAGACTGGGGCACATCCACGACTGCTGATGATCAAGCGAAGGGCACCGTTCGATGTTTGTGAAGTGGGCCTGGTGGGCACTGGGCGAGTCGGTCATG-9

TAGTTGGTCTAGTCGGTCATGCGGGCTACGGTTTGCTGGACGGCTTTATCACTAGACTGGGGCACATCCACGACTGCTGATGATCAAGCGAAGGGCACCGTTCGATGTTTGTGAAGTGAGCCTGGTGGGCACTGGGC-5

TAGTTGGTCTAGTCGGTCATGCGGGCTACGGTTTGCTGGAAGACGACATCACTAGACTGGGGCACATCCACGACTGCTGATGATCAAGCGGAGGACACCGTTCAATGTTTGTGAGGTAGGCCTGGTGGGCACTGGGC-6

TAGTTGGTCTAGTCGGTCATGCGGGCTACGTGTTTGCTGGACGGCTTTATCACTAGACTGGGGCACATCCACGACTGCTGATAATCAAGCGGAGGACACTGTTCAATGTTTGTGAGGTGGGCCTGGTGGGCACTGGGC-7

TAGTTGGTCTAGTCGATCATGCGGGCTACGTGTTTGCTGGACGGCTTTATCACTAGACTGGGGCACATCCACGACTGCTGATGATCAAGCGAAGGGCACCGTTCGATGTTTGTGAAGTGGGCCTGGTGGGCACTGGGC-8

TAGTTGGTCTAGTCGGTCATGCGGGCTACGTGTTTGCTGGACGGCTTTATCACTAGACTGGGGCACATCCACGACTGCTGATGATCAAGCGAAGGGCACCTTCGATGTTTGTGAAGTGGGCCTGGTGGGCACTGGGC-10

TAGTTGGTCTAGTCGGTCATGCGGGCTACGGTTTGCTGGACGGCTTTATCACTAGACTGGGGCACATCCACGACTGCTGATGATCAAGCGAAGGGCACCGTTCGATGTTTGTGAAGTGAGCCTGGTGGGCACTGGGC-5

TAGTTGGTCTAGTCGGTCATGCGGGCTACGGTTTGCTGGAAGACGACATCACTAGACTGGGGCACATCCACGACTGCTGATGATCAAGCGGAGGACACCGTTCAATGTTTGTGAGGTAGGCCTGGTGGGCACTGGGC-6

TAGTTGGTCTAGTCGGTCATGCGGGCTACGTGTTTGCTGGACGGCTTTATCACTAGACTGGGGCACATCCACGACTGCTGATAATCAAGCGGAGGACACTGTTCAATGTTTGTGAGGTGGGCCTGGTGGGCACTGGGC-7

TAGTTGGTCTAGTCGATCATGCGGGCTACGTGTTTGCTGGACGGCTTTATCACTAGACTGGGGCACATCCACGACTGCTGATGATCAAGCGAAGGGCACCGTTCGATGTTTGTGAAGTGGGCCTGGTGGGCACTGGGC-8

TAGTTGGTCTAGTCGGTCATGCGGGCTACGTGTTTGCTGGACGGCTTTATCACTAGACTGGGGCACATCCACGACTGCTGATGATCAAGCGGAGGACACCGTTCGATGTTTGTGAGGTGGACCTGGTGGGCACTGGGT-11

TAGTTGGTCTAGTCGGTCATGCGGGCTACGGTTTGCTGGACGGCTTTATCACTAGGCTGGGGCACATCCACGACTGCTGATGATCAAGCGGAGGACACCGTTCAATGTTTGTGAGGTGGGCCTGGTGGGCACTGGGC-12

TAGTTGGTCTAGTCGGTCATGCGGGCTACGGTTTGCTGGACGACGACATCACTAGACTGGGGCACATCCACGACTGCTGATGATCAAGCGAAGGGCACCGTTCGATGTTTGTGAAGTGGGCCTGGTGGGCGCTGGGC-13

TAGTTGGTCTAGTCGGTCATGCGGGTTACGGTTTGCTGGACGGCTTTATCACTAGACTGGGGCACATCCACGACTGTTGATGATCAAGCGAAGGGCACCGTTCGATGTTTGTGAAGTGAGCCTGGTGGGCACTGGGC-14

TAGTTGGTCTAGTCGGTCATGCGGGCTACGGTTTGCTGGAAGACGACATCACTAGACTGGGGCACATCCACGACTGCTGATGATCAAGCGGAGGACACCGTTCAATGTTTGTGAGGTAGGCCTGGTGGGCACTGGGC-6

TAGTTGGTCTAGTCGGTCATGCGGGCTACGTGTTTGCTGGACGGCTTTATCACTAGACTGGGGCACATCCACGACTGCTGATAATCAAGCGGAGGACACTGTTCAATGTTTGTGAGGTGGGCCTGGTGGGCACTGGTC-15

TAGTTGGTCTAGTCGATCATGCGGGCTACGTGTTTGCTGGACGGCTTTATCACTAGACTGGGGCACATCCACGACTGCTGATGATCAAGCGAAGGACACCGTTCGATGTTTGTGAAGTGGGCCTGGTGGGCACTGGGC-16

TAGTTGGTCTAGTCAGTCATGCGGGCTACGGTTTGCTGGACGGCTTTATCACTAGACTGGGGCACATCCACGACTGCTGATGATCAAGCGAAGGGCACCGTTCGATGTTTGTGAAGTGGGCCTGGTGGGCACTGGGC-17

TAGTTGGTCTAGTCGGTCATGCGGGCTACGTGTTTGCTGGACGGCTTTATCACTAGACTGGGGCACATCCACGACTGCTGATGATCAAGCGGAGGACACCGTTCAATGTTTGTGAGGTAGGCCTGGTGGGCACTGGGC-18

TAGTTGGTCTAGTCGGTCATGCGGGCTACGGTTTGCTGGACGACGACATCACTAGACTGGGGCACATCCACGACTGCTGATGATCAAGCGGAGGACACCGTTCGATGTTTGTGAGGTGGACCTGGTGGGCACTGGGT-19

TAGTTGGTCTAGTCGGTCATGCGGGCTACGGTTTGCTGGACGGCTTTATCACTAGGCTGGGGCACATCCACGACTGCTGATGATCAAGCGGAGGACACCGTTCAATGTTTGTGAGGTGGGCCTGGTGGGCACTGGGC-12

TAGTTGGTCTAGTCGGTCATGCGGGCTACGGTTTGCTGGACGACGACATCACTAGACTGGGGCACATCCACGACTGCTGATGATCAAGCGGAGGACACCGTTCGAGGTTTGTGAAGTGGGCCTGGTGGGCACTGGGC-20

TAGTTGGTCTAGTCGGTCATGCGGGCTACGGTTTGCTGGACGACGACATCACTAGTCTGGGGCACATCCACGACTGCTGATGATCAAGCGGAGGACACCGTTCAATGTTTGTGAAGTGGGCCTGGTGGGCACTGGGC-21

Figure S3. Partial 5′ and 3′ adjacent sequences in *C. nigoni* satellites of the 4_137_15 family. The adjacent sequences found at both ends of the satellites are compared. Only the first 100 bases are shown; similarity extends for about 500 bases. The data for the two long satellites in the X chromosome are unrelated and are not shown (X: 1543807 and X: 22932995). Related sequences in the corresponding region of *C. briggsae* are also included for comparison. The underlined region indicates the insertion of a microsatellite.

Adjacent 5′ Sequences

I: 2234586 GTTTCCCAGGAGGAGGCCAGCCAGATGCAGGTGGGCTAAGCCCCGCCCCTTTCTCTCACATATATAAGGGCAAGAAAAATCGGTAGGACACAGTTCGAT

I: 15299822 CGCAAGGGGGCGGAGTTTCCCCAGGGGCAGGTGTGCTAAACCCCGCCCCTCTCTCTCACGTATATAAGGGCAAGAAAAATCGGTAGGACACAGTTCGAT

II: 18190360 GCGTGTGCGTGTGCGTGTGCGTGTGCGTGAGTGTGCTAAGCCCCGCCCCTTTCTTTCACCTATATAAGGGGAAGAAAAATCGGTAGGACACAGTTCGAT

III: 1609305 GTTTCCCAGGGAGAGGTCAGCCAGATGCAGGTGTGCTAAGCCCCGCCCCTTTCTCTCACATATATAAGGGCAAGAAAAATCGGTAGGACACAGTTCGAT

III: 15035724 AGTTTCCCAGAAGAGGCCAGCCAGATGCAGGTGCGCTAAGCCCCGCCCCCTTCTCTCATATATATAAGGGCAAGAAAAATCGGTAGGACACAGTTCGAT

IV: 317549 GACGAAGATTCCCACAGGGCAAACTATCCAGGTGCGCTAAGCCCCGCCCACTTTTCCCCCTATATAAGGGCAAGAAAAATCGGTAGGACACAGTTCGAT

IV: 18108250 CAAAAGGAGAAAGGGGGCGGAGTTTACCACGTGCGCTAAGCCCCGCCCCTTTTTTTCACCTATATAAGGGAAAGAAAAATCGGTAGGACAAAGTTCGAT

IV: 18898545 GATGCAGTGTGTGTGTGCGCGCGCGCGTGAGTGTGCTAAGCCCCGCCCCTTTCTTTCACCTATATAAGGGGAAGAAAAATCGGTAGGACACAGTTCGAT

IV: 20134955 TACCACTCACATTTTTGGTTTATTGCCCCTTATGCA<u>**GTGTGTGTGTGTGTGTGTGTGTGTGTGTGTGTGT**</u>GAAGAAAAATCGGTAGGACACAGTTCGAT

V: 1300375 CTTCCCAGGAAGAGGTCAGCCAGATGCAGGTGTGCTAAGCCCCGCCCCTTTCTCTCACATATATAAGGGCAAGAAAAAGTCGGTAGGACACAGTTCGAT

V: 1305077 TTCCCACAGAAAGAGGCCAGATAGATGCAGGTGTGCTAAGCCCCGCCCCTTTCTTTCACATATATAAGGGCAAGAAAAATCGGTAGGACACAGTTCGAT

V: 20322659 TTTCCCTCAGGAAGACCCGCCAAACCCAGGTGTGCTAAGCCCCGCCCCTTTTTCTCGCATATATAAGGGCTAGAAAAAAGACGGAGGACACAGTTCGAT

91: 8959 CGCAAGGGGGCGGAGTTTCCCCAGAGGCAGGTGTGCTAAACCCCGCCCCTCTCTCTCGTATATATAAGGGCAAGAAAAATCGGTAGGACACAGTTCGAT

C. briggsae: Sequence 468

CACGGGAAGGATTAGGCCAGGTGCAGGTGGGCTAAGCCCCGCCCCCTTTTCTCCCAATA---CGGGCAA-AAAATTC--ATTTTAGGTGTTC

Adjacent 3′ Sequences

I: 2234586 ATGGGTTTTTGAAGGTGATCCCATTGGAAAAAAACCTTTTTTCCCTTTCTATGTATTGAGCCTCTACGTGGCGAAAGGGATCAACAGACTTTCTAGTC

I: 15299822 ATGGGGTTTTTGGTTGGTTTTTCCCCGGTAATAAATCATTTTCCCTTTCTATGTACTGAGCCTCTACGCGGCGAAAGGGATCAACAGACTCTATTTGT

II: 18190360 ATGGGGTTTTTGTTTGGTTTCTTCCCCCGGTAAAAAACCATTTTCCCTTTCTATGTACTGAGCCTCTACGTGGCGAAAGGGATCAACAGACTAAATTT

III: 1609305 ATGGGTTTTAGAAGGTGGTCCCATTGGTAAATAAACCTTTTTCCCTTTCTATGTACTGAGCCTCTACGTGGCGAAGGGGATCAACAGACT

III: 15035724 ATGGGTTTTTGAAGGTGATCCCATTGGTCAAATAAACCTTTTTTCCCTTTCTATGTACTGAGCCTCTACGTGGCGAAAGGGATCAACAGACTAGATTT

IV: 317549 ATGGGTTTTGGAAGGTGATCCCATTGGAAAATAAACCTTTTTTCCCTTTCATGTACTGAGCCTCTACGCGGCGAAAGGGATCAACAGACGAAAGTCTG

IV: 18108250 ATGGGTTTTTGAAGGTGATCCCATTGGTAAATAAACCTTTTTTCCCTTTCTATGTACTGAGCCTCTACGTGGCGAAAGGGATCAACAGACTACATTTG

IV: 18898545 ATGGGTTTGGAAGGTGTCCCATTGGTAAATAAACCTTTATTCCTTTTCATGTACTGAGCCTCTACGTGGCGAAAGGGATCAACAGACAAGAGTCTGGC

IV: 20134955 ATGGGTTTGGAAGGTGTCCCATCGGTAAATAAACCTTTTTCCTTTTCATGTACTGAGCCTCTACGTGGCGAAAGGGATCAACAGGCAAGAGTCTGGCT

V: 1300375 ATGGGTTTTGGAAGGTGATCCCATTGGTAAATAAACCTTTTACCCTTTCTATGTACTGAGCCTCTACGTGGCGAAAGGGATCAACAGACACAAGTCTG

V: 1305077 ATGGGTTTTGGAAGGTGATCCCATTGGTAAATAAACCTTTTTACCCTTTCTATGTACTGAGCCTCTACGTGGCGAAAGGGATCAACAGACACAAGTCT

V: 20322659 ATGGGTTTTCGGTTGGTTGTTTTCCCTGGTAAAAAAACCATTTTCCCTTTCATGTACTGAGCCTCTGCGTGGCGAAAGGGATCAACAGGCAAAAGTCT

91: 8959 ATGGGGTTTTTGGTTGGTTTTTCCCCGGTAATAAATCATTTTCCCTTTCTATGTACTGAGCCTCTACGCGGCGAAAGGGATCAACAGGCTCTATTTGT

C. briggsae:

Sequence 196

ATGGGTTTTTGAAGGTGATCCCATTGGTAAATAAACCTTTTTTCCCTTTCTATGTACTGAGCCTCTACGTGGCGAAAGGGATCAACAGACTACACTTGTCTGGCTA

Figure S4. Comparison of the consensus sequence of the *C. briggsae* proto-repeat and the corresponding satellites in *C. nigoni*. In the upper part is shown a comparison of the consensus of the proto-repeat in five *C. briggsae* 468 sequences and the corresponding 4_137_15 repeats in *C. nigoni*. The main difference is due to three insertions (dashes) in the second half of the satellite repeat, with a total of nineteen base pairs. The proto-repeat sequence was obtained from the 468 sequences in *C. briggsae* which have a match in *C. nigoni* (shown in Table 3). Below is shown the comparison with the consensus sequence of Family 33_107_4.

```
468  GA-GTTTGTGAG-TGG-CC-GTTGGGCACTGGGCTAGATTGGTCTAG-CGGTCATGCGG-CTACGG
137  GATGTTTGTGAGGTGGGCCTGGTGGGCACTGGGCTAGAT-GGTCTAGNCGGTCATGTGGGCTACGG
     ** ********* *** ** * ***************** ******* ******** ** ******

TTTGCTGGACG------TTCACTAGA-TGGGGCACAGCC—-GAC—---TATGGATCAGACG—A-GACA------
TYTGCTGGACGNCCTTTATCACTAGACTGGGGCACATCCACGACYGCTGAT-GATCAGACGGAGGACACCGTTC
***********       ******** ********* **  ***     ** ********* * ****
```

```
468  GAGTTTGTGAGTGGCCGTTGGGCACTGGGCTAGATTGGTCT-AGCGGTCA-TGCGGCT
107  GAAA--GT-AG-GCCAGTTGGATAATGGGCTAGCT-GGTCTGATCGGTCAGTTGGACT
     **    ** ** * * *****  * ********* * ***** * ****** *  * **

    ACGGTT--TGCTGGACGTTCACTAGATGGGGCACAGCCGACT-ATGGATCAGACG-AGACA

    TCGGTTCATACTGGAAGAGCT-TATCACTAGAACAG--GATTCAT--A-CTG-CGAATACT
     *****  * ***** *  *  **      * ****  ** * **  * * * ** * **
```

Figure S5. Dot-plot of a satellite in *C. nigoni* compared with its syntenic region in *C. briggsae.* Dot-plot of a satellite from *C. nigoni* at position V: 21478901 (vertical sequence), compared with its syntenic region in *C. briggsae* (horizontal sequence). The satellite belongs to the 11_155_10 family, only found in *C. nigoni*. It has 18 repeats of approximately 154 bases, with a total length of 2773 base pairs. A dimer of the same repeat is found in *C. briggsae* in a syntenic region. It appears that this is the first satellite in this family, grown from an ancestor genome region and starting with a sequence which has remained practically unchanged in *C. briggsae*. The 11_155_10 family appeared later by transposition of the first satellite to different regions of the *C. nigoni* genome.

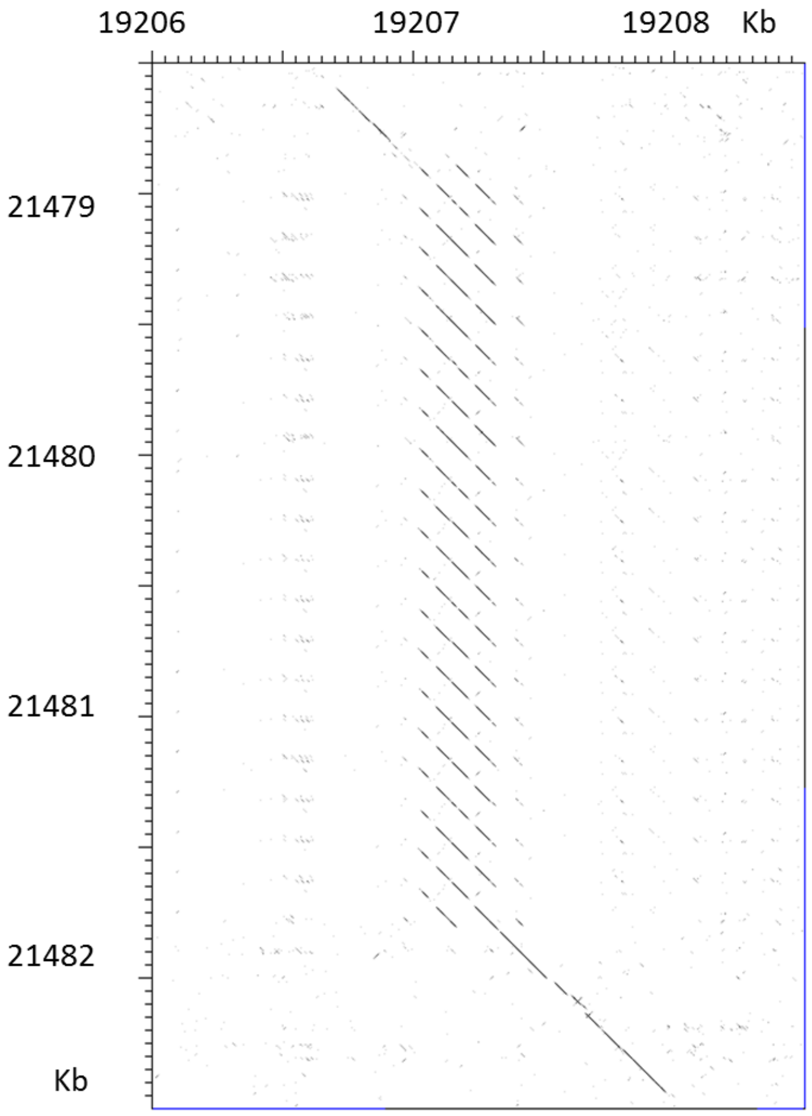

Table S1. Similarity score for *C. nigoni* satellites longer than 1 Kb. The table includes all satellites longer than 1 Kb and with a repeat length greater than 27 bases. The similarity score has been calculated excluding repeats with indels.

Scaffold or Chromosome	Genome coordinates		Satellite length	Repeat length	Number of repeats		Satellite Similarity score
	Start	End			All	Indels excluded	
1	3242756	3282669	39914	48	210	137	0.896278
5	1719441	1757592	38152	81	124	111	0.925738
6	17271358	17302865	31508	111	91	73	0.924825
18	204053	231501	27449	182	55	46	0.956702
35	49032	74462	25431	192	53	40	0.950561
32	49282	70957	21676	41	247	187	0.99114
11	120816	141068	20253	188	54	45	0.95355
5	1699323	1718838	19516	93	109	72	0.957006
6	8341177	8358233	17057	181	68	41	0.983955
18	251362	268384	17023	182	55	54	0.959356
6	12270073	12286545	16473	163	62	43	0.918418
16	251666	267948	16283	233	43	31	0.963576
4	9204470	9220499	16030	285	35	23	0.964126
5	13167743	13182458	14716	30	281	199	0.922648
25	166417	180955	14539	98	103	70	0.932536
49	20034	34499	14466	153	66	41	0.952835
1	9411059	9425226	14168	29	348	232	0.86014
6	19360309	19374241	13933	108	93	92	0.937785
11	206074	219688	13615	166	61	59	0.953582
6	18889764	18903081	13318	111	91	55	0.943734
6	16491027	16503925	12899	208	48	37	0.941449
5	20843135	20854783	11649	128	79	78	0.971775
6	8378462	8390089	11628	131	77	60	0.91237
12	10	11541	11532	118	86	78	0.977557
12	187689	199005	11317	180	56	41	0.936179
2	16299455	16310133	10679	167	60	55	0.905696
34	56599	67235	10637	29	279	270	0.95623
29	65142	75698	10557	182	55	52	0.949276
6	6747730	6758109	10380	182	55	48	0.928487
58	0	9907	9908	168	59	41	0.959795
6	6	9617	9612	30	305	280	0.850031
2	1567851	1577437	9587	86	112	68	0.966903
6	21768314	21777717	9404	122	72	59	0.866327
6	14992557	15001548	8992	111	81	81	0.961254
3	11045915	11054735	8821	167	53	34	0.967438
6	22915883	22924401	8519	152	56	50	0.940158
15	60346	68802	8457	202	42	26	0.943925
6	20521506	20529917	8412	172	51	42	0.911661
6	21768314	21776675	8362	122	66	54	0.869194
6	12402384	12410641	8258	192	43	42	0.931661
3	336602	344830	8229	78	105	67	0.958212
34	56599	64780	8182	29	240	235	0.957583
5	4955432	4963526	8095	119	68	60	0.868119
6	17913979	17922029	8051	122	66	64	0.923145
5	19149922	19157775	7854	167	47	45	0.92845
6	7591554	7599379	7826	182	43	42	0.941911
2	16420223	16427962	7740	180	43	30	0.925511
9	632036	639696	7661	133	57	56	0.919832
2	18024142	18031773	7632	87	88	66	0.977701

3	347934	355458	7525	78	96	60	0.956483
1	5950860	5958371	7512	29	259	241	0.897953
5	1404910	1412104	7195	90	80	79	0.962302
6	20429224	20436192	6969	213	33	22	0.930302
6	1428342	1434788	6447	30	209	205	0.900319
3	3448702	3455053	6352	29	226	194	0.941066
3	14779401	14785731	6331	87	73	52	0.971718
26	4958	11275	6318	29	237	145	0.838836
5	1691572	1697692	6121	102	60	60	0.928038
1	980454	986489	6036	85	71	71	0.957284
3	9751817	9757777	5961	28	222	185	0.921289
34	5	5854	5850	111	53	32	0.911436
4	734652	740425	5774	89	65	49	0.95233
2	18615090	18620837	5748	151	38	29	0.958547
5	4969233	4974945	5713	119	48	44	0.932288
2	18117046	18122676	5631	152	36	35	0.942297
4	11443103	11448675	5573	29	192	175	0.855573
6	15917889	15923427	5539	95	58	46	0.963506
4	14655257	14660765	5509	102	54	54	0.927187
11	365517	370891	5375	192	28	26	0.939062
16	23120	28455	5336	111	48	47	0.960308
34	70313	75518	5206	29	129	121	0.936759
3	8016282	8021462	5181	199	26	20	0.987834
6	1543807	1548937	5131	140	37	36	0.974694
3	14105547	14110662	5116	29	175	170	0.882415
6	16473154	16478169	5016	209	25	23	0.929749
21	214728	219630	4903	213	24	18	0.875868
6	20387999	20392749	4751	190	25	25	0.961123
34	56592	61248	4657	29	141	139	0.967987
22	193595	198246	4652	231	20	18	0.951674
9	179354	183949	4596	181	25	17	0.914852
6	22932995	22937528	4534	137	33	21	0.938918
6	21768313	21772827	4515	122	37	29	0.902312
26	4958	9452	4495	29	171	106	0.836376
2	6676617	6681090	4474	213	21	21	0.967572
3	225677	230139	4463	78	57	41	0.968147
2	14195688	14200146	4459	29	145	100	0.818386
6	13017053	13021383	4331	111	39	27	0.911022
98	3664	7989	4326	41	123	86	0.935493
4	12681143	12685447	4305	29	148	146	0.906851
11	529854	534077	4224	192	22	19	0.955409
93	5494	9670	4177	85	49	34	0.970333
5	752239	756404	4166	85	49	49	0.952648
6	2326064	2330158	4095	89	46	46	0.916583
5	1032718	1036615	3898	45	85	60	0.874919
2	14195667	14199521	3855	29	122	90	0.821752
5	19643748	19647570	3823	147	26	26	0.967068
14	311225	315023	3799	131	29	26	0.952868
5	12176093	12179781	3689	29	127	104	0.812721
2	6669721	6673341	3621	213	17	16	0.977152
11	536746	540349	3604	189	19	15	0.933216
4	10345968	10349558	3591	215	15	10	0.972024
15	400558	404039	3482	85	41	39	0.937255
98	8049	11503	3455	192	18	16	0.913681
2	6684467	6687902	3436	213	16	13	0.968907

6	4913121	4916552	3432	98	35	34	0.945748
5	5657525	5660899	3375	119	28	27	0.943307
5	9006072	9009443	3372	241	14	13	0.964393
6	6801704	6805010	3307	174	19	19	0.980283
24	107534	110761	3228	202	16	10	0.950862
34	56592	59775	3184	29	98	97	0.970316
2	10382010	10385106	3097	172	18	16	0.901541
1	9778646	9781712	3067	29	104	98	0.863056
4	8398500	8401560	3061	180	17	17	0.968518
4	19477877	19480888	3012	86	35	28	0.968852
1	13795197	13798157	2961	167	18	17	0.953211
3	13414994	13417950	2957	29	102	100	0.958416
4	10352689	10355602	2914	29	102	71	0.802401
11	356781	359660	2880	192	15	14	0.880089
6	14564174	14566974	2801	112	25	25	0.94117
1	7320186	7322921	2736	29	88	73	0.916444
4	18985987	18988703	2717	52	51	43	0.986881
5	22009352	22012064	2713	181	15	12	0.949886
6	12398575	12401228	2654	192	14	12	0.936132
6	20756081	20758711	2631	108	25	24	0.962203
3	10465163	10467749	2587	199	13	12	0.930164
1	11073707	11076288	2582	29	89	89	0.901589
6	1035693	1038273	2581	30	86	86	0.882839
34	70313	72879	2567	29	71	66	0.935311
2	8390221	8392774	2554	29	83	69	0.826257
18	183035	185585	2551	51	50	50	0.966515
5	11076793	11079337	2545	48	53	53	0.915094
4	538064	540539	2476	154	16	14	0.988297
6	20396638	20399108	2471	190	13	13	0.955016
3	10729592	10732003	2412	29	87	73	0.945135
12	88796	91190	2395	133	18	18	0.928907
5	4984140	4986519	2380	119	20	19	0.919931
148	8	2384	2377	108	22	22	0.943592
4	16426745	16429064	2320	29	64	46	0.872464
1	10480266	10482548	2283	29	79	75	0.857734
5	1757667	1759881	2215	42	57	47	0.932426
5	13391932	13394145	2214	29	79	54	0.879997
151	108	2316	2209	48	47	45	0.913524
4	317549	319751	2203	138	16	10	0.988621
34	78628	80828	2201	29	62	46	0.912732
3	15035724	15037916	2193	137	16	16	0.976561
153	12	2183	2172	167	13	13	0.936537
34	37105	39255	2151	29	68	67	0.876064
4	4844862	4847008	2147	29	74	74	0.90871
4	19571579	19573709	2131	152	14	12	0.997342
4	16436606	16438734	2129	56	38	38	0.914313
9	273931	276054	2124	193	11	11	0.967342
5	1757627	1759733	2107	42	54	45	0.941446
17	225326	227406	2081	174	11	9	0.637963
3	11428769	11430845	2077	29	70	44	0.898039
6	22798236	22800303	2068	172	12	10	0.953144
1	1735827	1737875	2049	103	19	13	0.458011
1	12183006	12185028	2023	29	60	46	0.848476
3	517682	519697	2016	155	13	13	0.962945
6	20656966	20658963	1998	60	24	16	0.733704

6	2059585	2061525	1941	30	61	39	0.963053
69	20946	22864	1919	192	10	8	0.936899
5	2494839	2496729	1891	63	30	30	0.947941
1	16131382	16133264	1883	131	15	11	0.994448
9	343676	345554	1879	193	10	9	0.966225
4	16430527	16432350	1824	29	60	40	0.871972
8	294917	296738	1822	103	15	9	0.514457
2	17968586	17970406	1821	140	13	13	0.991941
6	741620	743420	1801	72	25	25	0.74355
6	20656966	20658763	1798	60	22	16	0.733704
4	20121197	20122992	1796	105	17	12	0.959981
6	20982252	20984044	1793	112	16	16	0.948611
98	1286	3014	1729	192	9	9	0.938079
1	8485061	8486769	1709	29	56	36	0.909287
3	13175507	13177209	1703	29	58	57	0.915594
2	13644285	13645971	1687	29	61	46	0.907069
9	281801	283486	1686	193	9	8	0.968912
1	4937374	4939047	1674	29	60	47	0.894266
5	4965530	4967198	1669	119	14	12	0.906799
5	16997903	16999565	1663	29	56	55	0.951206
2	8514078	8515731	1654	29	57	57	0.886494
2	11544119	11545772	1654	29	53	37	0.822788
2	2418175	2419811	1637	29	54	34	0.881329
2	11606534	11608160	1627	29	44	34	0.873051
6	1827645	1829265	1621	30	54	50	0.831438
5	16160177	16161789	1613	29	56	49	0.866289
5	9443706	9445314	1609	201	8	8	0.96778
26	74423	76030	1608	29	46	38	0.879727
5	11727754	11729353	1600	29	47	35	0.884555
15	43485	45077	1593	201	8	6	0.9288
7	1244371	1245937	1567	29	53	52	0.909641
4	19656172	19657734	1563	153	11	10	0.980441
2	18062675	18064234	1560	156	10	9	0.977683
10	354612	356137	1526	29	53	40	0.870734
2	18199787	18201305	1519	270	5	3	0.774101
6	12394989	12396501	1513	108	14	14	0.915615
22	91111	92621	1511	29	39	31	0.857422
3	7231792	7233271	1480	29	51	51	0.928492
2	14833795	14835245	1451	103	15	10	0.505138
4	16505043	16506474	1432	29	50	31	0.908046
5	5568590	5570019	1430	119	12	11	0.865758
6	1031858	1033267	1410	30	46	44	0.867512
4	7776404	7777812	1409	29	46	40	0.865606
6	16478339	16479739	1401	175	8	8	0.996735
6	6806491	6807883	1393	174	8	8	0.968801
34	56592	57984	1393	29	48	48	0.9753
3	859214	860603	1390	151	5	3	0.988227
6	21106616	21108005	1390	85	17	14	0.931049
2	60964	62317	1354	172	6	4	0.971576
1	14174079	14175414	1336	134	9	6	0.780697
4	1570616	1571934	1319	38	29	23	0.871854
1	8110131	8111436	1306	29	43	41	0.953014
113	5220	6523	1304	29	28	23	0.973831
3	2199553	2200848	1296	181	5	4	0.859138
1	13281942	13283232	1291	191	5	3	0.587464

2	2933150	2934431	1282	130	9	7	0.989255
91	8959	10204	1246	138	9	8	0.978606
2	18803634	18804876	1243	156	8	6	0.991453
6	20986188	20987428	1241	124	10	10	0.978256
115	4053	5293	1241	124	10	10	0.973955
4	5299225	5300443	1219	174	7	7	0.978836
5	4564519	4565737	1219	174	7	7	0.987594
17	224341	225559	1219	174	7	7	0.986134
9	669242	670458	1217	174	7	5	0.970881
3	12836516	12837726	1211	29	39	33	0.92877
5	21193779	21194977	1199	60	20	19	0.983626
4	6235146	6236335	1190	29	39	37	0.885955
2	10873093	10874281	1189	198	6	6	0.968509
6	15890528	15891716	1189	108	11	11	0.934905
1	9013046	9014205	1160	29	39	38	0.920276
9	336771	337929	1159	193	6	6	0.972366
5	20901092	20902249	1158	144	8	5	0.723179
6	21909111	21910254	1144	114	10	7	0.983292
2	1026640	1027771	1132	151	5	3	0.635642
4	5593885	5595016	1132	29	38	37	0.833684
32	78351	79481	1131	188	6	4	0.98227
16	20653	21775	1123	111	11	8	0.985843
6	14754571	14755693	1123	66	17	17	0.915924
1	2234586	2235690	1105	138	8	8	0.995859
4	6735756	6736858	1103	29	37	36	0.937749
2	18785278	18786378	1101	156	8	6	0.961823
1	13708193	13709289	1097	137	5	3	0.801127
7	1055547	1056642	1096	39	28	17	0.893162
2	9491129	9492215	1087	29	38	36	0.904251
6	4946693	4947770	1078	98	11	10	0.954346
1	5896607	5897681	1075	29	33	26	0.782549
1	3300898	3301971	1074	183	5	3	0.676932
6	21905165	21906236	1072	115	10	9	0.947182
4	19568725	19569791	1067	152	7	5	0.996491
1	14737380	14738444	1065	299	5	3	0.892222
3	2402922	2403985	1064	288	3	2	0.764706
4	5293036	5294080	1045	174	6	6	0.980587
1	960970	962011	1042	214	5	4	0.753344
145	188	1222	1035	118	8	5	0.979661
4	16505666	16506694	1029	29	29	18	0.896627
5	20499818	20500845	1028	79	13	13	0.940495
1	10395281	10396296	1016	29	35	33	0.811825

Table S2. Similarity score for *C. briggsae* satellites longer than 1 Kb.. The table includes all satellites longer than 1 Kb and with a repeat length greater than 27 bases. The similarity score has been calculated excluding repeats with indels. Note that many of these satellites are in unplaced positions (Scaffold 8).

Chromo-some or Scaffold	Genome coordinates		Satellite length	Repeat length	Number of repeats		Satellite Similarity score
	Start	End			All	Indels excluded	
8	2170104	2180111	10008	182	54	40	0.962487
8	2103266	2112894	9629	60	158	131	0.945685
8	2199997	2209451	9455	163	58	55	0.941055
X	3045691	3054535	8845	60	153	130	0.93372
8	2321206	2328889	7684	122	63	58	0.953752
X	14040228	14047758	7531	163	45	39	0.972579
X	16109363	16116768	7406	111	79	51	0.980121
8	2344365	2351322	6958	167	41	33	0.964737
8	804369	811274	6906	111	59	50	0.897167
X	3045691	3052109	6419	60	113	96	0.933733
X	16146022	16152312	6291	163	38	35	0.949064
IV	7589171	7595262	6092	285	21	20	0.931844
X	3045691	3051554	5864	60	104	89	0.941624
V	8340032	8345792	5761	48	120	118	0.918502
8	2522264	2527502	5239	154	34	31	0.94295
8	1078745	1083943	5199	42	138	100	0.954485
I	7899138	7904303	5166	231	22	21	0.850065
8	2533049	2538143	5095	182	29	25	0.97177
X	19959934	19964757	4824	122	37	35	0.959627
IV	11945633	11950251	4619	51	88	84	0.916941
8	2558278	2562856	4579	60	76	65	0.927393
8	2562976	2567371	4396	163	27	21	0.924433
8	2585912	2590279	4368	182	24	23	0.968959
X	13547944	13552016	4073	163	25	22	0.970148
II	15598505	15602483	3979	153	26	26	0.980908
I	14801646	14805605	3960	83	46	37	0.97998
8	2621036	2624944	3909	163	24	18	0.912587
X	3045691	3049519	3829	60	67	59	0.943477
X	7201627	7205394	3768	139	28	23	0.975394
X	8369545	8373300	3756	163	23	15	0.948583
IV	11945633	11949304	3672	51	72	69	0.926851
V	11910849	11914434	3586	30	78	47	0.868599
X	686383	689852	3470	30	93	61	0.773571
IV	7589030	7592450	3421	285	12	12	0.92628
I	8250796	8254185	3390	231	14	13	0.980464
X	13716813	13720134	3322	111	30	27	0.881622
V	9760420	9763729	3310	48	68	66	0.917495
X	6239151	6242425	3275	182	18	16	0.973321
X	12939311	12942475	3165	167	18	12	0.970846
X	13732376	13735483	3108	111	28	25	0.874692
8	2395480	2398576	3097	163	19	18	0.95632
X	19959934	19962949	3016	122	23	22	0.968964
8	2767237	2770239	3003	163	18	15	0.919291

8	2745422	2748354	2933	163	18	16	0.952011
X	9546152	9549065	2914	182	16	15	0.958277
8	2751808	2754717	2910	182	16	11	0.956044
8	2770365	2773205	2841	139	22	14	0.983661
8	2310363	2313136	2774	154	18	17	0.944296
X	1419498	1422171	2674	30	86	85	0.850096
X	7567531	7570094	2564	154	15	14	0.939108
I0	5750	8308	2559	93	27	25	0.959761
IV	13221500	13224049	2550	102	25	24	0.974851
I	7895292	7897833	2542	231	11	11	0.95162
8	2809919	2812459	2541	231	11	10	0.961776
V	3707592	3710110	2519	81	31	24	0.978708
X	15239981	15242488	2508	163	15	12	0.940881
III	3285776	3288266	2491	161	12	8	0.553537
X	9600527	9603014	2488	131	19	17	0.97695
8	2825814	2828215	2402	80	30	27	0.988889
8	2833543	2835885	2343	81	29	22	0.971015
8	2634783	2637119	2337	146	16	16	0.967047
X	4738997	4741332	2336	146	16	15	0.97382
I	7899059	7901371	2313	231	10	9	0.852493
I	7980044	7982352	2309	231	10	8	0.961451
8	2817968	2820276	2309	231	10	8	0.833436
8	1092024	1094306	2283	60	37	34	0.954288
8	2820587	2822866	2280	285	8	7	0.949652
8	2848413	2850676	2264	81	28	23	0.979831
III	4173513	4175758	2246	228	8	5	0.803202
IV	17418034	17420243	2210	47	47	47	0.97276
X	9562051	9564243	2193	180	10	6	0.746834
8	2865120	2867299	2180	81	27	21	0.969273
I	14796851	14799018	2168	83	26	22	0.980737
I	7975875	7977953	2079	231	9	8	0.961245
8	2878773	2880851	2079	231	9	8	0.956298
X	6695536	6697604	2069	139	16	10	0.9838
8	2874275	2876318	2044	146	14	13	0.972134
8	2885499	2887533	2035	81	25	16	0.988066
8	2858230	2860249	2020	81	25	17	0.954328
X	6201110	6203112	2003	182	11	11	0.973893
8	2898716	2900711	1996	285	7	7	0.947424
X	15631137	15633091	1955	163	12	10	0.935651
X	11637013	11638965	1953	163	13	9	0.948648
V	4462792	4464688	1897	119	16	11	0.962923
8	1856834	1858717	1884	81	23	16	0.993827
X	12939311	12941193	1883	167	11	7	0.968824
IV	10412802	10414575	1774	200	9	7	0.989206
8	2576703	2578471	1769	163	11	9	0.976369
II	896003	897770	1768	50	23	14	0.876032
X	4734682	4736432	1751	146	12	10	0.975038
8	2911492	2913233	1742	30	50	34	0.810893
X	13555360	13556994	1635	163	9	8	0.969033
X	15239981	15241612	1632	163	10	9	0.942286
X	18107349	18108968	1620	108	15	14	0.896622
V	18177738	18179345	1608	33	35	34	0.941014
I	8301079	8302660	1582	99	15	14	0.854516
V	1994803	1996378	1576	63	25	25	0.893021
X	738360	739925	1566	72	22	17	0.63281

I0	5568	7113	1546	93	16	15	0.968049
X	925328	926860	1533	81	19	12	0.985036
IV	14538632	14540130	1499	28	52	38	0.885863
8	1895208	1896661	1454	135	8	6	0.670663
X	684778	686217	1440	30	43	37	0.819486
X	9565574	9567000	1427	182	8	7	0.963719
X	14787786	14789186	1401	34	42	28	0.880278
X	2983358	2984745	1388	42	39	27	0.937141
IV	10412142	10413509	1368	200	5	3	1
IV	15948808	15950173	1366	50	19	16	0.939788
X	16127489	16128819	1331	111	12	11	0.978378
I	13797729	13799057	1329	134	10	8	0.533309
V	861344	862649	1306	45	29	29	0.933881
X	14040212	14041515	1304	163	8	7	0.958711
X	19950324	19951580	1257	129	8	5	0.66359
8	1497513	1498748	1236	111	13	9	0.979313
I	13837044	13838263	1220	145	6	5	0.696491
X	18105086	18106274	1189	108	11	11	0.901235
I	7973125	7974279	1155	231	5	4	0.962482
8	1704757	1705896	1140	285	4	3	0.965692
X	4707068	4708206	1139	163	7	5	0.964008
8	2393099	2394229	1131	163	5	4	0.968643
X	15626899	15628029	1131	163	5	4	0.968643
II	896003	897118	1116	50	19	14	0.876032
IV	3987627	3988737	1111	29	38	26	0.94992
II	1168951	1170047	1097	162	7	6	0.760643
V	879970	881059	1090	45	25	17	0.935948
X	7391158	7392239	1082	180	6	4	0.962861
8	1895208	1896256	1049	135	5	3	0.593398

Table S3. Octamer repeats (ARATTCWG) in *C. nigoni* and *C. briggsae*

Species	**Number of repeats**	**AAATTCTG**	**AAATTCAG**	**AGATTCTG**	**AGATTCAG**	**Total**	**ARATTCWG**
C.nigoni	**4-7**	**275/313**	**1169/1238**	**1755/1548**	**8/4**	**6310**	**6129**
	**>7**	**245/227**	**1492/1629**	**1528/1280**	**6/8**	**6415**	**7026**
C.briggsae	**4-7**	**109/160**	**1273/1166**	**676/616**	**3/2**	**4005**	**4326**
	**>7**	**70/52**	**862/773**	**368/319**	**3/3**	**2450**	**2762**

The table shows the number of sequences with more than three repeats of different variants of ARATTCWG. The number of direct and reverse repeats are separated with a dash. The "Total" column gives the sum of the perfect repeats given in the previous four columns. Comparison with the ARATTCWG column demonstrates that these satellites have a strong bias to present stretches of identical repeats. Note that *C. nigoni* significantly favors the AGATTCTG repeat. These sequences are part of large families, with consensus repeats which incorporate these octamer sequences. They are completely absent in other *Caenorhabditis* (*C. elegans*, *C. remanei* and *C. brenneri*). An additional feature of these octamer repeats is the low frequency of AGATTCAG, which has the same CG% as the frequent AGATTCTG. It appears that the AGATTCAGAGATTCAG… sequence is deleterious, probably by generating an unwanted signal for transcription factors binding to this sequence.

Table S4. All satellite families

Family	Repeat	Nr satellites	Satellites Cnigo	Satellites Cbrig	Score	Consensus sequence
1	29	72	71	1	0.758817	GGAATCGgAGGATCGGAGCTGgTGGAGGT
2	28	38	22	16	0.600936	TAGGTCATGaCCTAGAAAaTcCAAAAAT
3	30	21	14	7	0.726062	AACTAtCACCTCtGAACCTACTcCAGtTtC
4	35	15	9	6	0.606667	ATTACTGTAGggGAAAatattcGAAAATgAaAAgg
5	85	13	13	0	0.745262	GGATATATGGGGATATATCAagGGCATCtGGAtATGGgaGnACCATATCaaGGCATcAGAGAATCTTgCGATTacTCTgGCATCc
6	32	13	9	4	0.602259	tTTTgCTACTGCTnaGCAGTanCaAAaTncGa
7	33	12	8	4	0.632576	tTTTcAAAATCGGAATATTCcGAAATTcCgAAn
8	29	12	12	0	0.895507	CGGCTATCGCGACtATCAGACCATATAAG
9	67	10	10	0	0.990713	CTGTCGCGCTGGCCGTCTATGCGATGGAGGAGACGGCTGCCGCGACAGTGGTGTGTGTCCaTTACTG
10	81	9	0	9	0.972565	TGAGAGGATTGTTGTGAAAATTAATAAAGAcCCTTTTTCGTTGTTTTTGCATcAATTCTCCCCAGAGTTTGAAGGAACCAG
11	46	9	2	7	0.698068	TGgCCTAGAAAactcAatTtGCnAAAGTTAGgCCAcCAtgTcaAAA
12	52	8	0	8	0.929487	TTCTGGGATTTTGGGGATATTCACGTGATTCACAGAATTCGAACGGTTCGAG
13	45	6	4	2	0.765012	GTCTGCGTCTCTCTTGCcGccGcGAGAGACGCAGcGTGTCTCGTT
14	36	6	6	0	0.965432	CCACAGCAGAATCCAGGACCTTCTCACAGCCAGCTa
15	30	6	2	4	0.677037	TTCTAATAgCTcAAAAaATagGTCAGaAng
16	52	5	5	0	0.749444	CCTCCTAaACTTCGAACGGTGTCCTCAGAATGATCCTCAGtAtccgAAgATA
17	57	5	5	0	0.631034	GGCCTAGAAAACaCACcanGtgcATgTcaggnCatTTCtaggcCattttgAaGTggT
18	57	5	5	0	0.955172	AAAATTTTTTTGATTTTTTCAAAAAATCGATATTCCAATCAAGAGGAAAAAAATcAG
19	41	5	2	3	0.765079	CGGaAAATcGngTTCtCGAAtTTTTtCtCGActTTtTTTTt
20	33	5	5	0	0.658571	tTgTcCGGATTCcGGATTcCGggAAAaTnTntt
21	31	5	5	0	0.869792	GGGGACGGGGctCGTCCCCGTCCCTTTTtTc
22	78	4	4	0	0.835764	tTCTCCCGACTCaacTAcCAATTAAAGTACTGgGAACCCATTACCCAACTGcTAgcCTTATCTGAAgTcaaGCTGCcC
23	73	4	1	3	0.691111	GTAGTTTGTaGTCTAGCAgaCCCAAatgAcGcATTTCtaaTaCacacatGaTgGGtCTGCTAATGTTtctatt

24	65	4	4	0	0.630837	TTACTGAGGCTaAgGAgaTTctTgTAGTTtGTAGtcccTaAGCCTCAGTAAAcGcgcaccacgtT
25	60	4	0	4	0.925926	CaACATCtTCAGCTGAGACCTCGACAATtGCCACcTCGACCGCTGAGACtAcTACAGTTC
26	57	4	4	0	0.984405	AAAAAATTgTTATAAGGAGTTATGGACCAAAACGTACCAAAAAATGGGTAAATTTTC
27	48	4	2	2	0.884259	GTAGTgGAgCTGGATGGtTCgGCTGTCGTGCTCGAcGAtGGgACTTCA
28	44	4	4	0	0.954545	AACTACTCATAGCGATGAGCTAGTTAACaAGGAATaCAGATTTc
29	41	4	4	0	0.645995	gAAggaTTCTagtTTTGcCGTcAatcTTCTgaaTTTGCGgT
30	38	4	4	0	0.627083	CTTgaCgCatTtCtaGTGCcTTGACGCaTTTcTngTat
31	35	4	4	0	0.653509	ccttgtgatttTcaaatatTTtaccttacagTaat
32	35	4	2	2	0.65015	AAAtattcgagaaaggAAaggAttActGtagggag
33	33	4	1	3	0.760943	CCCTTCTATTgTTCCAAGntaccTCtCGGAgGG
34	32	4	2	2	0.617845	TAAGAAAaatTCTcActAgGAAacaTccTcAc
35	32	4	2	2	0.631944	cGACgTTTcAGAAcTgcgAaATTTcaaAACca
36	29	4	4	0	0.722222	TAcAGTtAGAAgGCgACCAAgtAaAagCA
37	28	4	3	1	0.782567	CCcTTCTATtAGAgGTAcCTcCaGAgTG
38	28	4	1	3	0.746032	aTTTTTCACttcGATTTTTTtgtcCCca
39	87	3	1	2	0.795658	TCGGGTGCCAcgaTTTTcGCcaCTTTCTTTGcTcgTTTCGAaCcagTCTCTGGTTGAGCatCTTtTGCTGCAGCTgt GGTAGCtGTa
40	45	3	1	2	0.812346	CAACtACTGAAGCTcCtACCACnAcCACtAtGGAaCCTTCCACCa
41	41	3	3	0	0.751323	cGGAATTTCGGaATtTcGcAAaaTTCCGATTTTcAaAAaTT
42	35	3	2	1	0.701754	cTaCAGTAATCCTTTTCATTTtCAAnTAnTTnAAA
43	38	3	2	1	0.836257	TTTTTGATATTATGATAAcCATtTTgTtAGTTgAcaAC
44	35	3	3	0	0.974603	CAGCAAtTTGCTGGTTTTTTCCTTATGCAGAAAAT
45	35	3	0	3	0.707937	tCTACAGTAATCcTtTTnaTTcTCAAAcAntTnTT
46	29	3	2	1	0.670034	ACcGGACGTTttAGAACcAgTATTTCGCA
47	32	3	2	1	0.626263	AAATTcnGAAnTTcAaAAAtTCgAAnATTnAg
48	32	3	1	2	0.652778	TTCTcAgtTTCTGAaatTCnGAaatTCTcAgA
49	32	3	1	2	0.708333	ACTGTCTGCTTCAatttTCtctTGTttnTCTA
50	30	3	3	0	0.703704	aAcTCTCTCGCTTcTCTAAcCGTCtgcgta
51	99	2	1	1	0.838384	CAACTCCAGCAAAGGcTACaCCaAAGgCcaCAGCTGCTAAGAAGGCcGaTTCTTCATCGGACGATTCTTCT GACGACGAGAAaAAaCCTGCgGCTAAAa

52	98	2	2	0	0.904762	CAGAGTGATTTTTTGCAAATTTTaGCAATTTTCAAGCTTCAaGACTaTGTAAAATCCCCATCaACCCTTCT TTTcAGGTGAACCATCGCaCACACcGC
53	97	2	1	1	0.821306	GTTGGCgCCAAAGGcACgAACAGATcCcaAAGACGCTTTATTTTCAGATGCAGaAGATGTTCCGAAcCCA GATGaTCTcAAAaCCAcTTGCACTGGC
54	93	2	1	1	0.842294	AGTGAAATGACATCTGAAGCaTCTaCTTCTACTGTAACTCaATCaACATCATCAGAaCCaTGcGAGACGG AcAGCACAGaATCAAGccCATCG
55	90	2	0	2	0.940741	ACTGGATATCCAGTaGAGTTTCCAaACGTTGGGTACTCGGTAGAGTTTCCAAAGaaTGGCAAGTCCGTA GAATTTCCAGCTTGATAAGAG
56	86	2	0	2	1	TCAATTTTGATCCCTCAAAATGTCATATACGTCTTTAGAAATGTCATTTTTAAAGCCGTAAAATGTCAT ATATGACCCTAAAAATG
57	83	2	0	2	1	CTCCGCCCACTTTTCAGAAAGTGGGCGTGGTCTTGAAAATTCGAGATTGGGCGAAGCTTAAAACTAGC TCAAACTCGAATAGG
58	75	2	0	2	0.637778	TTcCGGATTTTCaGAATcTCGGAATATcCCgAaTTTcaAaaaTgCGAAATgTTaGAATATcCCaATcTTcAgAAt
59	72	2	2	0	0.814815	AGGAACATcAGaGGGAACACGCCGAGGATCCCGAaGacGAGATGGcAATGGAGCAaaCCAAGgATCTGC TCa
60	69	2	2	0	1	GTTTCTTCTGAACGGGCTCTTGACTTTCTTGCTCTATCTGAAGATTTCCGATGATGATACTGTTCCTCG
61	69	2	2	0	0.884058	TTACGGAAGGAAAGAGATGCTGAATACCAAaGGATCAGATTGAGTCAGGAAACcCcaGAACAAGCaAA c
62	63	2	1	1	0.89418	TGGACGCTTCTTCTCCTCCTCaGCTCCTTGaGTTCCCTTcGGCcGATCCTCaGCAGCTCTAGC
63	61	2	2	0	0.978142	GCCTCTCATTGTGAAGCTTCGATAACAAGGCCAACTCAGTTCTTTTTTGATAAcTCGGCTA
64	60	2	2	0	0.711111	aAAATTCAAAaTTGacCaAacTagGTCGTGTTTAGGCTCATTTTGAAGcTCTAAACgcaA
65	59	2	0	2	0.629943	TTTCTAGGCCACTaTGcTAAacTGGCcTAGaAAACaTaCTcAGaTGAATATcaGGaCag
66	57	2	2	0	0.929825	TCAATAGTCTAAAATCGAAAAAACCTGACAAGCCATCCGAAAGTcCcATCAATGCTc
67	54	2	0	2	0.604938	ACGACAAAATGcaCAaaAATTTcaaATTTTTccGaAATTTTGCAtaCAgATTTG
68	54	2	0	2	0.925926	AAGCGCATCTCcTCCGGCGATGGGACCTCCAATTGAGCTGGaCCATGGTgTTGG
69	53	2	1	1	0.647799	TCTTGTGATCCATGGCCaGCagAcATGTaTcTTTcTCcGGATTaCaaTAgGGA
70	51	2	1	1	0.79085	AGGATCTGAAGaACCAAAgCCATCCGATgCTTCTTCacAAGcAaCAGTaGG
71	50	2	2	0	0.733333	TGTGCATTTTGgCGTCaAATTcaGaCGTATTACcCAAAAATTcGAAAAac
72	48	2	1	1	0.680556	TACTGTAgTTTTTAgGATTcCaTTTTTcAAacTGAcAAAtATGAGGAT
73	48	2	2	0	0.75	GTTCgTCGGAaCAAcTTACAGaTCTTcTTGGACaAaCCAAAGGAcCAa
74	48	2	2	0	0.944444	GGGAATCTGAAATcTaGAAAAGTGGGAAGCCTGGAATAAAGAAAAGTA
75	48	2	0	2	0.916667	GACGGCTCCACAACTTATcGTCCATGGGAGACAACCCCATACCCaGAc

76	46	2	2	0	0.623188	CCTAGAAAaCAcAcCAGGGgCAcgTgAgaCCATTTTaAaacGATGG
77	45	2	1	1	0.911111	GGATTCGGATTCGAaAAGCGAGCTCTcGACGGGCTTGAcGGTGCC
78	45	2	0	2	1	TGGCCTAGAAACCACAACTCTTAAAAATTAGGTCATCATCGTAAC
79	44	2	0	2	0.670455	ATTTTCAGAcaCAAAATGgAtGaTTTTTAaGCTGAAAATcgTag
80	44	2	0	2	0.640152	gTCATTTTTGAcCcAATaTAaGaCCATTTaTaAGCCAAacTaGA
81	43	2	2	0	0.709302	gCTTaAAATgGGTCaAaATAGGCTgAAAATGACaTgAAAATTa
82	43	2	2	0	0.600775	GGaTgTTTAcAAgCcAAAAaTGAaaTTTTTgGgCaTaTTTTGa
83	42	2	1	1	0.666667	TcGGaGGAGCACCAAtggGAGGAgCTAGcACgATGACcGCcG
84	42	2	2	0	0.746032	CCGAgcCGGAATaTTTCCaAAGacTTTTTTCGGAATGAAATc
85	42	2	1	1	0.873016	CCAGGAAAGCGGTCAATGGCcTACGGACGaCAAGGaTTCCGa
86	42	2	0	2	0.968254	TCCACaAGTACCTCGACTGAATCCACCTCCACATCGACCGAG
87	40	2	0	2	1	CCGTTTTCCGTTTTCCGCTTGGTTTCTAGAGACCTTTTTT
88	39	2	1	1	0.606838	aAaTGCGTCAAaGcACtGAAAAAtGCGCCAAgAaAcCcA
89	38	2	0	2	1	AAGGTTACTGTAGCTCAAATATAAAACCAGCGAGATGT
90	38	2	0	2	1	AAATGCGTCATGGATCGAACATAGCGTCAAGGCACAGA
91	37	2	1	1	0.675676	AGTAATCCTTgTcATTTgTTTTataATTTcTTCtTAC
92	37	2	2	0	0.626126	TTCCATTGGGAaTTTCcaTTcTgaTTcTGaTgTCCCg
93	37	2	2	0	1	AATTGCGAAATTTCAAATTTCCCGCGAAATCCCTTAA
94	37	2	2	0	1	TGGGCGGAGAATCAAAGACGCATACTTTTCTGAAATA
95	36	2	2	0	0.962963	CAGCTCCTCGAGCTTTTCCTCGGCCTGATCCTTaAA
96	36	2	1	1	0.62963	TCaTCaTCcACgTCTTCacCaTCCcCTTCcAcTTCT
97	35	2	1	1	0.619048	AATAGAAGcCCacCCTCTAATTGaaacCCaCCTcT
98	35	2	1	1	0.714286	gAAAATagAAACGATTAaTGTAAaGaAAAATGTTa
99	34	2	2	0	0.666667	AAaaTTCTgGAaTTCTaGAAATTCgGAAATTTcG
100	34	2	2	0	0.666667	TTTTTCaAAATTcTGGaAAaaTCgAAAAATcGAa
101	34	2	1	1	0.960784	GAATAAGTAGTACCAAaTCGATACTACTTACTCG
102	34	2	2	0	1	AACCGTCTGCTGCCGCCCTTATGCTTTTTCATTT
103	33	2	1	1	0.676768	aAAgcCTGAaATTCTGAAATCCTAtGAtTTaTG
104	33	2	2	0	0.79798	GGATTCCGGAAAATcAGTTTCATTCcacAaTCC

105	33	2	1	1	0.79798	GCAGCACCAgcAGCAGGAGGACcTgGACGTTGg
106	33	2	1	1	0.676768	TGCTCCATTCACacTcCCAAcaCaTCCAACaaT
107	33	2	0	2	0.636364	TTcGaAaCCaGTCAaTTCaAAacTAcGTGACAC
108	32	2	1	1	0.604167	AAAATTAGGTCatgACGaAAaAaAAaTAcGAa
109	32	2	0	2	0.666667	AAAATGACCCaaAAATcAtGAAAtAacGGCTG
110	32	2	2	0	0.708333	AATCATGATTTTgAGCcTATTTTacaCcTGAa
111	32	2	1	1	0.75	ATTTTAGAACCGCGACGTTTCaaAACcacAaC
112	32	2	0	2	0.625	aAAATTcTGAaATTCCcAAAcTaTaaAATTCa
113	31	2	1	1	0.61828	AAaTTGCCTAAAaATacCTAaaTTgGcCTGa
114	30	2	1	1	0.65	tTTTcAaTTTCcCgCCAAAaAAtTTCAAAA
115	30	2	1	1	0.866667	CTGGAGCAGCcTCGACTGGTTTCTCTTcaA
116	30	2	1	1	0.777778	CTGGAAACaATGTAaTCGAcGCAACGAgaT
117	30	2	0	2	0.822222	GCAACTTGCTAAAaCagTTTCATGCATGaG

Table S5. Comparison of conserved satellites The table shows the internal similarity of satellites which are conserved in both species. The consensus identity was determined without allowing any gaps. In a few cases the repeat length differs; the consensus identity is given for the common region of both satellites. The similarity column is a measure of the internal variability of repeats in the satellite, excluding indels. High values indicate frequent internal recombination events which result in a more uniform sequence of the satellite. The identity value gives the percentage of bases which are identical in the consensus sequence of the two satellites under comparison. The values are high, as expected from the short evolutionary time which separates the two species; they give a measure of the variability of satellites on an evolutionary time scale, to be compared with young satellite families which have recently appeared (described in section 3.5). The results shown in this table demonstrate that satellites have evolved continuously from its precursor in the ancestor species. The internal similarity usually differs in each of the pairs of satellites compared, which indicates different internal mutation and recombination events. However the average value of internal similarity is practically the same in both species (0.84 and 0.82), which demonstrates similar mutation and recombination rates in the two species. The identity value is always greater than 70% (average value 86.6%), which indicates a limited rate of change in conserved non coding regions of the genome in these two species. Further analysis shows that the internal similarity of a few satellites is low (<0.6), which indicates the presence of a substantial number of internal mutations. In such cases the presence of mutations has prevented the growth and homogenization of the satellite by internal recombination, whereas the consensus sequence between the two species has been only partially altered.

Repeat length	Family	Position		Length of satellite (bp)		Internal similarity		Consensus identity (%)
		Cbrig	Cnigo	Cbrig*	Cnigo	Cbrig	Cnigo	
204/186	53-204-2	I: 444908	I: 964546	605	577	0.837	0.752	71.6
30	46-29-3	I: 836260	I: 1451403	91	301	0.559	0.831	86.7
96	53-97-2	I: 7509548	I: 8147444	499	577	0.819	0.840	93.7
134	25-133-5	I: 13797729	I: 14895575	1339	808	0.533	0.683	78.4
145	65-147-2	I: 13837044	I: 14847597	1230	727	0.696	0.847	77.2
171/180	59-182-2	II: 2215316	II: 2435627	861	715	0.597	0.780	75.0
36	4-35-15	II: 5745429	II: 6577733	154	179	0.784	0.728	83.3
29	1-29-72	IV: 3987627	IV: 4916893	1111	704	0.950	0.920	93.1
285	18-285-6	IV: 7589171	IV: 9204470	6092	16030	0.932	0.964	93.3
51	69-53-2	IV: 10561916	1V: 11786331	205	154	0.700	0.747	88.2
51	70-51-2	IV: 11945633	IV: 13155553	4619	715	0.917	0.939	94.1
102	77-102-2	IV: 13221500	IV: 14655257	2550	5509	0.975	0.927	91.2
28	2-28-38	IV: 14258926	IV: 15837967	228	288	0.924	0.796	89.7
28	2-28-38	IV: 14538632	IV: 16320440	1499	175	0.886	0.985	93.1
28	2-28-38	IV: 14737707	IV: 16883773	227	618	0.952	0.765	96.4
155	12-154-9	IV: 17359147	IV: 19656172	467	1563	0.991	0.980	85.2
45	40-45-3	V: 861344	V: 1032718	1306	3898	0.934	0.875	86.7

63	62-63-2	V: 1994803	V: 2494839	1576	1891	0.893	0.948	96.8
119	14-120-8	V: 4462792	V: 5568590	1897	1430	0.963	0.866	88.2
46	-	V: 5220569	V: 6422607	242	507	0.460	0.518	78.3
87	39-87-3	V: 9396539	V: 10714938	340	340	0.873	0.758	89.7
48	27-48-4	V: 9760420	V: 11076793	3310	2545	0.917	0.915	89.6
30	115-30-2	V: 11910849	V: 13167743	3586	14716	0.869	0.923	96.7
144/152	64-152-2	V: 18059266	V: 19898626	440	609	0.991	0.719	78.5
30	3-30-21	X: 684778	X: 714431	3470	874	0.774	0.865	90.0
72	-	X: 738360	X: 741620	1566	1801	0.663	0.744	88.9
30	3-30-21	X: 1050257	X: 1035693	636	2851	0.705	0.883	87.1
30	3-30-21	X: 1419498	X: 1428342	2674	6447	0.850	0.900	93.3
30	3-30-21	X: 1954829	X: 2008544	182	92	0.674	0.733	76.7
103	2-103-24	X: 2273535	X: 2379617	412	310	0.965	0.845	92.2
42	85-42-2	X: 4463821	X: 4792450	286	268	0.873	0.746	92.9
131	34-131-4	X: 9600527	X: 8378462	2488	11628	0.977	0.912	74.0
163	3-163-19	X: 11637013	X: 12270073	1953	16473	0.949	0.918	70.6
167/191	-	X: 12939311	X: 13708063	3175	22651	0.969	0.895	79.6
111	20-111-6	X: 13716813	X: 14564174	3322	2801	0.882	0.941	77.5
34	101-34-2	X: 14787786	X: 15698891	1401	613	0.880	0.880	91.2
108	17-108-7	X: 14923035	X: 15877458	884	973	0.984	0.923	95.4
45	77-45-2	X: 15041559	X: 16024900	520	529	0.599	0.615	88.9
111	16-111-7	X: 16109363	X: 17271358	7406	31508	0.980	0.925	85.2
30	116-30-2	X: 16372277	X: 17528602	91	91	0.970	0.748	83.3
108	36-108-4	X: 18103109	X: 19360309	6000	13933	0.893	0.899	94.4
122	15-122-7	X: 19959934	X: 21768314	4824	9404	0.960	0.861	74.6
33	105-33-2	X: 20220852	X: 22095960	232	166	0.969	0.733	90.9
132	69-141-2	X: 21287252	X: 23577050	641	528	0.874	0.874	87.1

*The length of the longest satellites in *C. briggsae* is often underestimated, since they contain many unassigned bases (Ns). Also in several cases there are related satellites in unplaced regions in *C.briggsae* (Scaffold 8), which could be part of satellites shown in the table.

Table S6. Syntenic regions of *C. briggsae* satellites in the genome of *C. nigoni.* All satellites in *C. briggsae* which present a significant degree of synteny with *C. nigoni* are shown in the table. The region of synteny, the length of the aligned region and the % of identical bases are given. "Conserved" indicates that a satellite detected by SATFIND is found in the syntenic position. A complete description of conserved satellites is given in Table S5.

***C.** briggsae* satellites					Syntenic region in *C.nigoni*		
Chromosome	Start	Length	Repeat Length	Number repeats	Start alignment	Length alignment	% identity
I	444908	605	204	3	964546	577	Conserved
I	836260	91	30	3	1451403	301	Conserved
I	1039828	378	107	3	1663574	343	0.71
I	2169589	947	136	6	2789243	653	0.74
I	2263356	147	43	4	2879582	145	0.77
I	2430170	217	86	3	3021977	182	0.58
I	4673106	178	35	3	5513197	158	0.60
I	5230889	170	45	3	6046584	164	0.81
I	5516172	352	35	8	6312396	337	0.75
I	7058256	71	28	3	7857669	68	0.86
I	7257364	550	28	3	7929513	561	0.76
I	7273404	128	51	3	7950106	128	0.74
I	7509548	499	96	6	8147444	577	Conserved
I	8709303	437	123	3	9105459	295	0.94
I	9023816	195	72	3	9435722	183	0.95
I	10289544	236	38	3	10686279	213	0.68
I	10767547	145	30	4	11257673	142	0.84
I	11163043	252	46	5	11974867	475	Conserved
I	11285079	466	102	3	12035601	458	0.63
I	11369835	389	173	3	12170117	387	0.80
I	12533182	342	60	5	13671390	209	0.54
I	12735303	429	139	3	13369233	-375	0.54
I	13234184	448	49	8	14196416	-306	0.50
I	13510150	604	137	3	14557237	582	0.71
I	13561965	595	203	3	14615011	525	0.63
I	13612630	706	147	5	14711195	-697	0.58
I	13640864	543	180	3	14731622	476	0.68
I	13797729	1329	134	10	14894340	538	Conserved
I	13837044	1220	145	6	14847597	727	Conserved
I	14167129	352	112	3	15171418	-392	0.57
I	14702489	402	133	3	15975792	405	0.77
I	14796851	2168	83	26	16071806	641	0.49
I	14801646	3960	83	46	16072432	1737	0.50
II	811071	340	30	6	831101	-203	0.48
II	909776	578	147	3	1047043	586	0.75
II	1042304	726	138	3	1144808	522	0.66
II	1045793	747	138	4	1141713	-722	0.64
II	1167436	479	162	3	1437417	480	0.68
II	1168951	1097	162	7	1438626	983	0.61
II	1230024	434	144	3	1065222	401	0.60
II	1462722	343	45	3	1689945	-271	0.53

II	1845928	379	52	3	1957521	405	0.72
II	2215316	861	171	3	2435627	715	Conserved
II	2235552	753	192	3	2461655	774	0.74
II	2258951	93	31	3	2485092	95	0.72
II	2276847	325	62	3	2502311	325	0.64
II	2760354	246	35	5	3120119	248	0.64
II	3055226	445	111	4	3442052	206	0.74
II	4848744	131	44	3	5653002	129	0.80
II	5184725	226	28	6	6029474	222	0.71
II	5343353	257	86	3	6187613	263	0.72
II	5745429	154	36	3	6577733	179	Conserved
II	10903300	206	35	5	11885097	204	0.76
II	11995661	109	31	3	13196685	106	0.76
II	12852945	191	63	3	13841644	178	0.73
II	13027524	496	28	16	14309635	435	0.74
II	15358252	82	33	3	17645581	-79	0.83
II	15381715	371	40	3	17314343	383	0.61
II	15489410	359	74	5	17872153	352	0.78
II	15785027	939	73	14	18231357	528	0.55
II	16019191	624	73	3	18493712	613	0.81
II	16022900	213	71	3	18497683	241	0.65
II	16115360	752	30	22	18607547	415	0.75
III	552255	235	52	5	509980	221	0.70
III	839686	463	182	3	801820	468	0.63
III	1000816	452	150	3	1040531	439	0.73
III	1079697	426	135	3	1753681	-429	0.57
III	1238127	373	30	3	1588269	-360	0.55
III	2088416	488	162	3	2430691	-481	0.53
III	2609874	413	110	4	2588963	-404	0.62
III	2716607	708	73	4	2826410	681	0.77
III	3285775	803	161	5	3557140	806	0.71
III	3308862	321	41	5	3576542	321	0.65
III	4653235	574	143	4	5441827	559	0.73
III	5853405	327	30	3	6577668	317	0.80
III	5955140	107	35	3	6670026	95	0.72
III	7749537	109	34	3	8418522	102	0.76
III	8351142	140	35	4	9115128	133	0.77
III	9929082	96	38	3	10639814	93	0.76
III	10968826	524	32	3	11559634	119	0.52
III	11593484	359	101	3	12354921	344	0.75
III	11834731	563	187	3	12828263	-553	0.68
III	12096026	555	191	3	12895294	573	0.67
III	12153455	594	137	3	12958178	472	0.62
III	12321275	274	73	3	13215181	-277	0.66
III	12607141	395	32	13	13430066	384	0.65
III	12680946	690	38	5	13515662	678	0.75
III	12691736	690	38	5	13515662	678	0.75
III	12831294	566	195	3	13619608	542	0.63
III	13013679	415	135	3	13926764	417	0.74

III	13019757	438	155	3	13932870	432	0.77
III	13045727	443	147	3	13956760	413	0.60
III	13047071	603	151	3	13957569	595	0.71
III	13157248	510	135	4	14047725	499	0.72
III	13507669	520	195	3	14516462	-516	0.72
III	13551056	622	69	3	14471403	-391	0.48
III	13911255	563	42	14	14798958	429	0.59
III	14152437	116	29	4	15050092	111	0.85
III	14330540	222	101	3	15169879	224	0.74
IV	47238	108	35	3	505327	92	0.63
IV	761323	158	31	3	867993	169	0.74
IV	1094299	123	30	3	1229413	122	0.73
IV	1606345	289	46	6	1780599	274	0.74
IV	1788904	335	101	3	2060414	349	0.71
IV	3696039	145	45	3	4566919	155	0.72
IV	3987627	1111	29	38	4916893	704	Conserved
IV	7292680	702	61	3	9536832	-37	0.62
IV	7589171	6092	285	21	9204470	16030	Conserved
IV	10412142	1368	200	5	11608007	1444	0.80
IV	10412802	1774	200	9	11608740	1787	0.81
IV	10550080	147	33	3	11776312	151	0.72
IV	10561916	205	51	4	11786331	151	Conserved
IV	11211702	109	36	3	12490309	99	0.93
IV	11945633	4619	51	88	13155553	715	Conserved
IV	13221500	2550	102	25	14655257	5509	Conserved
IV	13915346	859	102	5	15551859	818	0.59
IV	14244100	113	28	4	15851607	-113	0.89
IV	14258926	228	28	8	15837967	288	Conserved
IV	14446230	259	57	3	16222239	261	0.76
IV	14477714	111	34	3	16248790	111	0.67
IV	14538632	1499	28	52	16320440	175	Conserved
IV	14737707	227	28	7	16883773	618	Conserved
IV	15205405	569	189	3	17662517	-552	0.56
IV	15624326	512	31	11	18046350	415	0.58
IV	15719872	645	161	4	18131870	641	0.62
IV	15886086	449	149	3	18188071	-454	0.58
IV	15948760	951	50	19	18396841	876	0.65
IV	15948808	1366	50	19	18397661	619	0.52
IV	16097875	397	132	3	18599933	333	0.71
IV	16190174	732	183	4	18682811	341	0.57
IV	16213051	546	193	3	18704716	505	0.63
IV	16490562	196	42	4	19018067	-199	0.62
IV	16548726	549	183	3	18962918	-445	0.63
IV	16845107	513	171	3	19338349	-495	0.55
IV	16983032	321	129	3	19536493	326	0.78
IV	17359147	467	155	3	19656172	1563	Conserved
V	100217	169	48	4	106123	166	0.76
V	861344	1306	45	29	1032718	3898	Conserved
V	1560478	288	41	3	2074794	277	0.73

V	1994803	1576	63	25	2494839	1891	Conserved
V	2356808	473	172	3	2884084	385	Conserved
V	2691436	149	46	3	3199592	144	0.74
V	3601350	631	54	3	4729255	636	0.65
V	3876530	424	41	3	4791926	416	0.73
V	4462792	1897	119	16	5568590	1430	Conserved
V	4814691	313	90	3	5981555	322	0.79
V	4816693	313	90	3	5983629	343	0.83
V	5220569	242	46	4	6422607	507	Conserved
V	5377298	112	45	3	6589524	109	0.94
V	8798333	486	129	3	10026493	430	0.63
V	9396539	340	87	3	10714938	340	Conserved
V	9402342	340	87	3	10714938	340	Conserved
V	9760420	3310	48	68	11076793	2545	Conserved
V	11910849	3586	30	78	13167743	14716	Conserved
V	13747224	664	166	4	15040146	686	0.68
V	13966499	71	30	3	15260431	61	0.70
V	14332414	481	156	3	15623120	505	0.80
V	15410401	142	54	3	16779895	139	0.88
V	17942084	273	91	3	19684359	248	0.65
V	17949525	215	31	3	19690152	210	0.59
V	18192726	597	145	3	19965831	144	0.71
V	18271515	115	33	3	20142735	112	0.78
V	18338526	139	32	4	20253953	137	0.81
V	18363067	668	162	3	20276894	635	0.72
V	18400024	340	28	10	20312732	339	0.65
V	18447694	439	32	3	20219799	-363	0.54
V	18457463	355	32	5	20209415	-323	0.57
V	19456719	458	152	3	19780190	-450	0.56
X	738360	1566	72	22	741620	1801	Conserved
X	1419498	2674	30	86	1428342	6447	Conserved
X	1954829	182	30	5	2008544	92	Conserved
X	2273535	412	103	4	2384119	582	Conserved
X	2455078	346	33	9	2634141	361	0.72
X	2681141	487	54	9	2871915	483	0.73
X	2973968	454	28	5	3155566	439	0.57
X	3235964	305	36	5	3431553	298	0.90
X	4165269	127	36	4	4481735	123	0.73
X	4463821	286	42	3	4792450	268	Conserved
X	5900861	116	29	4	6332161	115	0.79
X	8933519	550	171	3	9709656	604	Conserved
X	9176030	293	30	3	9956537	312	0.57
X	9600527	2488	131	19	8378462	11628	Conserved
X	9744801	306	51	6	10414968	294	0.61
X	11637013	1953	163	13	12270073	16473	Conserved
X	13385661	586	196	3	14174569	578	0.70
X	13716813	3322	111	30	14564174	2801	Conserved
X	13732376	3108	111	28	14564174	2801	Conserved
X	14249130	259	46	3	15145245	245	0.74

<table>
<tr><td>X</td><td>14787786</td><td>1401</td><td>34</td><td>42</td><td>15698891</td><td>613</td><td>Conserved</td></tr>
<tr><td>X</td><td>14818166</td><td>128</td><td>32</td><td>4</td><td>15753640</td><td>126</td><td>0.83</td></tr>
<tr><td>X</td><td>15041559</td><td>520</td><td>45</td><td>7</td><td>16024900</td><td>529</td><td>Conserved</td></tr>
<tr><td>X</td><td>15370889</td><td>439</td><td>140</td><td>3</td><td>16361147</td><td>431</td><td>0.81</td></tr>
<tr><td>X</td><td>16109363</td><td>7406</td><td>111</td><td>79</td><td>17271358</td><td>31508</td><td>Conserved</td></tr>
<tr><td>X</td><td>16372277</td><td>91</td><td>30</td><td>3</td><td>17528602</td><td>91</td><td>Conserved</td></tr>
<tr><td>X</td><td>17078624</td><td>961</td><td>192</td><td>5</td><td>18306423</td><td>468</td><td>0.69</td></tr>
<tr><td>X</td><td>17244294</td><td>911</td><td>182</td><td>5</td><td>16407927</td><td>237</td><td>0.51</td></tr>
<tr><td>X</td><td>17777973</td><td>117</td><td>29</td><td>4</td><td>19041914</td><td>117</td><td>0.73</td></tr>
<tr><td>X</td><td>18103109</td><td>865</td><td>108</td><td>8</td><td rowspan="3">19360309</td><td rowspan="3">13933</td><td rowspan="3">Conserved</td></tr>
<tr><td>X</td><td>18105086</td><td>1189</td><td>108</td><td>11</td></tr>
<tr><td>X</td><td>18107349</td><td>1620</td><td>108</td><td>15</td></tr>
<tr><td>X</td><td>18117753</td><td>133</td><td>33</td><td>4</td><td>19383466</td><td>131</td><td>0.78</td></tr>
<tr><td>X</td><td>18999819</td><td>528</td><td>173</td><td>3</td><td>20655581</td><td>359</td><td>0.81</td></tr>
<tr><td>X</td><td>19277140</td><td>433</td><td>54</td><td>8</td><td>20982252</td><td>1793</td><td>Conserved</td></tr>
<tr><td>X</td><td>20218604</td><td>120</td><td>30</td><td>4</td><td>22094022</td><td>206</td><td>Conserved</td></tr>
<tr><td>X</td><td>20220852</td><td>232</td><td>33</td><td>7</td><td>22095960</td><td>166</td><td>Conserved</td></tr>
<tr><td>X</td><td>20242164</td><td>154</td><td>42</td><td>3</td><td>22116849</td><td>150</td><td>0.73</td></tr>
<tr><td>X</td><td>21287252</td><td>641</td><td>132</td><td>5</td><td>23577050</td><td>528</td><td>Conserved</td></tr>
</table>

Table S7. Syntenic regions of *C. nigoni* satellites in the genome of *C. briggsae.* All satellites in *C. nigoni* which present a significant degree of synteny with *C. briggsae* are shown in the table. The region of synteny, the length of the aligned region and the % of identical bases are given. "Conserved" indicates that a satellite detected by SATFIND is found in the syntenic position. A complete description of conserved satellites is given in Table S5.

***C. nigoni* satellites**					**Syntenic region in *C.briggsae***		
Chromosome	Start	Length	Repeat Length	Number repeats	Start alignment	Length alignment	% identity
I	980454	6036	85	71	462158	1147	0.48
I	1153761	332	129	3	600011	321	0.77
I	1451403	301	30	8	836178	296	0.73
I	1735827	2049	103	19	836260	91	Conserved
I	1831524	550	183	3	1193207	543	0.8
I	1932518	866	289	3	1299564	855	0.74
I	1959353	421	171	3	1324968	421	0.7
I	2234586	1105	138	8	1610403	1033	0.5
I	2297177	599	30	5	1662158	590	0.62
I	2305854	518	172	3	1672487	478	0.61
I	2362425	128	42	3	1737752	139	0.7
I	2525428	310	60	6	1892873	317	0.63
I	2559551	380	42	9	1927361	392	0.65
I	2609139	527	173	3	1974513	520	0.69
I	3039923	740	145	5	2447109	731	0.69
I	3300898	1074	183	5	2847437	1048	0.79
I	3309715	748	183	5	2855731	410	0.75
I	3902998	661	137	3	3285934	519	0.54
I	5950860	7512	29	259	5143252	2070	0.45
I	5962850	105	35	3	5149600	99	0.73
I	6620527	671	33	5	5957569	628	0.63
I	7320186	2736	29	88	6620511	468	0.44
I	8147444	577	96	6	7509548	499	Conserved
I	9051475	175	71	3	8650669	172	0.93
I	9063000	315	88	3	8662205	312	0.95
I	9460212	142	35	4	9049340	152	0.65
I	9464593	104	35	3	9054647	102	0.76
I	9778646	3067	29	104	9372679	531	0.44
I	10956693	305	101	3	10549144	300	0.72
I	11253493	107	36	3	10763133	104	0.71
I	11682206	475	28	14	11163043	252	Conserved
I	12183006	2023	29	60	11385132	1369	0.47
I	12506638	578	151	3	11643543	575	0.63
I	12738796	676	135	4	11816131	642	0.62
I	13218051	693	173	4	12896305	-473	0.49
I	13276470	434	144	3	12829002	-439	0.77
I	13278099	435	145	3	12831051	-427	0.62
I	13675357	448	137	3	12538144	267	0.81
I	13708193	1097	137	5	13050772	-1102	0.63
I	14174079	1336	134	9	13212105	573	0.64
I	14203937	588	138	3	13228003	-444	0.73

I	14472746	520	103	3	13387427	-552	0.55
I	14481482	361	60	3	13378020	-352	0.69
I	14737380	1065	299	5	13646836	548	0.66
I	14765390	545	181	3	13676528	505	0.71
I	14847597	727	145	5	13837044	1230	Conserved
I	14894340	538	134	4	13797729	1339	Conserved
I	14895575	808	135	6	13797060	-652	0.51
I	15057138	417	122	3	13943513	-304	0.68
I	15088840	330	102	3	13906454	-391	0.57
I	15424688	397	126	3	14281826	393	0.68
I	15648447	184	64	3	14377339	199	0.62
I	15737052	294	77	4	14449710	256	0.6
I	15861811	340	59	3	14638638	-339	0.67
I	15930096	530	64	7	14652082	508	0.64
I	15931264	571	64	3	14653448	476	0.59
I	15959317	191	32	6	14680383	193	0.68
I	16131382	1883	131	15	14839775	793	0.63
I	16304738	291	75	4	15033919	287	0.64
II	699454	308	102	3	529567	309	0.73
II	748526	130	32	3	594850	-126	0.65
II	1200951	503	169	3	975352	-499	0.76
II	1243411	649	169	3	936218	-498	0.51
II	1278670	549	182	3	1290905	545	0.61
II	1405243	365	44	3	1421316	376	0.66
II	1421590	542	135	4	1152083	542	0.73
II	1606142	409	122	3	1256697	414	0.75
II	1730448	439	96	3	1569664	-418	0.59
II	1910515	549	192	3	1770230	427	0.57
II	1978665	391	40	3	1794915	383	0.6
II	2227274	112	42	3	2052241	115	0.66
II	2278532	527	181	3	2110977	230	0.67
II	2418175	1637	29	54	2207763	945	0.43
II	2435627	715	180	4	2215316	861	Conserved
II	2506929	401	96	5	2282276	414	0.68
II	2522098	191	29	6	2317400	189	0.59
II	2534548	201	31	3	2330019	199	0.78
II	2933150	1282	130	9	2635132	671	0.46
II	3099891	712	128	5	2742193	444	0.62
II	3661286	503	53	5	3236635	517	0.66
II	5166739	496	165	3	4725279	-518	0.65
II	6026667	157	56	3	5181944	153	0.72
II	6577733	179	36	5	5745429	154	Conserved
II	6676617	4474	213	21	5807717	3120	0.45
II	9344099	900	29	31	8397564	588	0.45
II	10382010	3097	172	18	9454804	886	0.55
II	10544329	167	29	5	9607286	166	0.67
II	11160077	106	35	3	10231787	111	0.79
II	11544119	1654	29	53	10609652	733	0.44
II	11623616	141	35	3	10680450	106	0.9

II	12106367	222	44	5	11100512	226	0.73
II	12842982	322	129	3	11661781	343	0.68
II	13157273	121	30	4	11961855	118	0.8
II	13223364	88	29	3	12024314	82	0.69
II	13372209	313	28	9	12135518	313	0.79
II	13452200	794	187	3	12205806	784	0.74
II	13838124	209	67	3	12851268	183	0.55
II	14833795	1451	103	15	13391941	133	0.48
II	15667127	572	42	3	14041139	-449	0.49
II	15802629	654	31	3	14128549	-244	0.52
II	15860773	631	49	12	14155664	429	0.5
II	15892802	201	71	3	14188356	206	0.75
II	17065805	153	38	5	14797822	-154	0.76
II	17851436	211	30	3	15462294	209	0.76
II	17968586	1821	140	13	15536099	1256	0.48
II	18117046	5631	152	36	15671812	364	0.48
II	18137313	348	73	5	15688529	295	0.68
II	18167216	440	63	3	15718132	-463	0.55
II	18187439	222	74	3	15738809	217	0.82
II	18225780	639	73	3	15778150	612	0.84
II	18489060	221	74	3	16014409	219	0.7
II	18584933	235	42	5	16094248	234	0.7
II	18601169	666	129	3	16109457	676	0.67
II	18785278	1101	156	8	16240328	843	0.49
II	18803634	1243	156	8	16240328	843	0.49
II	18915506	366	45	3	16338187	55	0.56
III	225677	4463	78	57	286610	578	0.46
III	336602	8229	78	105	397010	298	0.47
III	347934	7525	78	96	397010	298	0.5
III	505643	562	191	3	548664	465	0.5
III	916732	746	188	3	894884	757	0.69
III	1421949	593	193	3	1403181	-556	0.62
III	1579176	395	54	3	1247182	-410	0.57
III	1737647	585	195	3	1095584	-545	0.63
III	1740014	610	202	3	1093497	-510	0.56
III	1900799	556	184	3	1822908	552	0.65
III	2106774	488	172	3	2018511	479	0.76
III	2162688	129	43	3	2397740	-127	0.62
III	2225695	427	145	3	2300611	-399	0.64
III	3081091	429	198	3	2955840	377	0.6
III	3250916	763	187	3	3112357	-740	0.61
III	3606413	166	53	3	3342477	154	0.7
III	5342248	247	35	6	4530479	87	0.55
III	5357975	779	223	3	4542766	898	0.77
III	5458862	535	35	15	4669495	382	0.65
III	5723938	152	56	3	4927253	147	0.91
III	6559146	107	35	3	5832305	104	0.81
III	6751420	108	37	3	6021034	109	0.75
III	7084720	260	35	3	6350999	109	0.49

III	7840483	117	42	3	7126453	43	0.53
III	7861340	115	33	3	7151938	106	0.79
III	8353576	169	60	3	7685576	166	0.83
III	9072307	192	30	3	8297705	201	0.61
III	9751817	5961	28	222	9015791	743	0.59
III	11807432	355	49	6	11127490	352	0.74
III	12046096	115	38	3	11349887	112	0.75
III	12395400	415	138	3	11636737	381	0.68
III	12512291	590	214	3	11744385	577	0.65
III	12995965	405	138	3	12187646	401	0.64
III	13037879	551	184	3	12235032	543	0.64
III	13156344	169	42	4	12388402	-171	0.63
III	13213627	549	184	3	12322625	-546	0.58
III	13259509	551	184	3	12467473	210	0.6
III	13312208	537	174	3	12529177	495	0.66
III	13396955	848	282	3	12581061	-715	0.56
III	13769533	716	145	5	12895876	689	0.62
III	14039680	537	137	3	13149544	537	0.72
III	14052745	405	135	3	13161973	389	0.68
III	14186358	672	163	3	13276926	719	0.68
III	14191595	485	162	3	13284773	480	0.7
III	14263875	644	154	3	13787288	-636	0.75
III	14607729	542	135	4	13373807	-550	0.59
III	14779401	6331	87	73	13895433	2312	0.47
IV	249776	322	107	3	312300	217	0.67
IV	317549	2203	138	16	242079	-812	0.45
IV	499596	85	29	3	39710	82	0.65
IV	649220	915	365	3	1555942	832	0.69
IV	885394	481	137	3	777084	475	0.71
IV	1565517	493	113	3	1376801	414	0.63
IV	1570616	1319	38	29	1382414	293	0.61
IV	2448776	406	54	5	2120757	401	0.62
IV	2569615	282	60	3	2201911	265	0.66
IV	4000877	654	31	3	3319951	-386	0.49
IV	4634238	308	102	3	3745001	223	0.72
IV	4864740	110	37	3	3939409	90	0.73
IV	4867478	133	61	3	3941882	117	0.62
IV	4916893	704	29	21	3987627	1111	Conserved
IV	5293036	1045	174	6	4324379	737	0.49
IV	5299225	1219	174	7	4324379	737	0.48
IV	5417853	340	28	12	4442462	329	0.5
IV	5593885	1132	29	38	4598921	367	0.46
IV	7317083	492	44	5	6122198	486	0.52
IV	7408095	644	31	3	6184191	217	0.49
IV	8006093	196	33	3	6774199	193	0.95
IV	9204470	16030	285	35	7589171	6092	Conserved
IV	11443103	5573	29	192	10171033	340	0.44
IV	11786331	154	51	3	10561916	205	Conserved
IV	12607207	220	54	3	11304916	214	0.9

IV	12681143	4305	29	148	11368871	498	0.42
IV	12912186	172	34	5	11561407	163	0.71
IV	13155553	715	51	14	11945633	4619	Conserved
IV	13780308	301	101	3	12447637	318	0.64
IV	14166025	575	129	3	12839885	318	0.81
IV	14655257	5509	102	54	13221500	2560	Conserved
IV	15837967	288	29	10	14258926	228	Conserved
IV	16320440	175	29	6	14538632	1449	Conserved
IV	16883773	618	28	20	14737707	227	Conserved
IV	17031913	756	149	5	14868778	742	0.76
IV	17760723	559	192	3	15445405	526	0.74
IV	17992540	319	42	6	15568067	304	0.57
IV	18149534	310	145	3	15735327	-325	0.59
IV	18929950	531	174	3	16429774	537	0.64
IV	19058390	472	161	3	16458384	-250	0.55
IV	19475017	317	43	5	16917382	-307	0.53
IV	19477877	3012	86	35	16915311	-1027	0.48
IV	19505237	510	129	3	16947374	472	0.74
IV	19756282	687	46	13	17077326	318	0.45
V	813914	98	33	3	654808	88	0.81
V	1032718	3898	45	85	861344	1306	Conserved
V	1364305	201	63	3	1309006	196	0.77
V	2370584	797	102	8	1830846	-341	0.55
V	2494839	1891	63	30	1994803	1576	Conserved
V	2884084	385	128	3	2356808	473	Conserved
V	2926247	291	99	3	2403386	301	0.67
V	3260405	579	193	3	2514883	544	0.61
V	3383485	121	30	5	2811824	119	0.78
V	4803934	672	224	3	3887366	534	0.63
V	4816478	342	102	3	3906456	364	0.71
V	5139345	578	150	3	4111588	514	0.58
V	5169158	307	102	3	4139476	294	0.63
V	5454653	241	30	6	4359093	239	0.73
V	5568590	1430	119	12	4462792	1897	Conserved
V	5657525	3375	119	28	4531381	1434	0.49
V	5877677	114	28	3	4685336	113	0.66
V	5985362	304	101	3	4818397	323	0.65
V	6319149	503	103	5	5096754	227	0.57
V	6391694	414	103	5	5181048	207	0.57
V	6422607	507	46	7	5220569	242	Conserved
V	6657153	347	29	11	5432946	332	0.69
V	7034794	169	60	3	5850097	85	0.47
V	8171158	317	129	3	6887704	311	0.77
V	8642896	518	129	5	7379984	500	0.68
V	9204324	174	50	3	7957652	47	0.44
V	9443706	1609	201	8	8163178	651	0.47
V	10714938	340	87	3	9396539	340	Conserved
V	11076793	2545	48	53	9760420	3310	Conserved
V	11727754	1600	29	47	10414579	1004	0.44

V	12176093	3689	29	127	10868776	212	0.44
V	13167743	14716	30	281	11910849	3586	Conserved
V	14498866	91	30	3	13228871	90	0.78
V	16997903	1663	29	56	15521924	-274	0.4
V	19729967	456	152	3	17996377	429	0.65
V	19732885	449	152	3	17999095	176	0.59
V	19888114	486	32	6	18069488	-469	0.54
V	19898626	609	152	4	18059266	440	Conserved
V	19920036	139	32	3	19473015	-139	0.68
V	19945639	933	152	4	18169921	751	0.61
V	20122085	113	32	3	18252832	108	0.75
V	20201345	524	32	3	18466767	-532	0.56
V	20243213	160	32	5	18327169	166	0.66
V	20247677	583	32	17	18331570	325	0.63
V	20249355	138	32	4	18332881	135	0.76
V	20282900	449	64	7	18368917	202	0.53
V	20327228	97	32	3	18417094	93	0.8
V	21068811	287	32	5	18848239	284	0.73
V	21179252	315	129	3	18954559	312	0.85
V	21300928	513	129	5	19058794	509	0.77
X	741620	1801	72	25	738360	1566	Conserved
X	1439261	220	33	6	1425019	221	0.58
X	1658835	133	33	4	1621228	133	Conserved
X	1827645	1621	30	54	1790233	574	0.5
X	2005517	211	30	7	1951978	210	0.81
X	2008544	92	30	3	1954829	182	Conserved
X	2097987	438	72	6	2037815	394	0.54
X	2384119	582	46	10	2273535	412	Conserved
X	2579203	277	46	5	2394890	269	0.7
X	2723561	216	67	3	2553194	191	Conserved
X	3656901	508	36	5	3764614	-527	0.62
X	4792450	268	42	3	4463821	286	Conserved
X	6043615	579	220	3	5602149	597	0.74
X	6047739	121	30	3	5606420	118	0.87
X	6592472	79	33	3	6139898	76	0.89
X	6801704	3307	174	19	6255765	1519	0.49
X	7591554	7826	182	43	6773926	1406	0.48
X	7680304	166	33	5	6862689	153	0.56
X	7781354	157	36	5	6947638	158	0.91
X	7783994	85	33	3	6949763	82	0.95
X	8341177	17057	181	68	9584889	7552	0.48
X	8378462	11628	131	77	9600527	2488	Conserved
X	9709656	604	207	3	8933519	560	Conserved
X	12270073	16473	163	62	11637013	1953	Conserved
X	13017053	4331	111	39	12327502	56	0.57
X	14399013	253	36	7	13544922	55	0.48
X	14564174	2801	112	25	13716813	3322	Conserved
X	14754571	1123	66	17	13914057	1046	0.7
X	14992557	8992	111	81	14116369	1622	0.49

X	15698891	613	34	18	14787786	1411	Conserved
X	15877458	973	108	9	14923035	884	Conserved
X	15890528	1189	108	11	14927891	37	0.53
X	15900116	973	108	9	14927891	37	0.53
X	15905258	541	108	5	14927891	37	0.55
X	16024900	529	45	8	15041559	530	Conserved
X	16491027	12899	208	48	15463683	684	0.67
X	17182087	307	102	3	16063811	288	0.7
X	17271358	31508	111	91	16109363	7406	Conserved
X	17528602	91	30	3	16372277	91	Conserved
X	17913979	8051	122	66	16756593	2731	0.54
X	18889764	13318	111	91	17660653	485	0.5
X	19138627	643	41	3	17870881	525	0.59
X	19188671	652	30	4	17924006	643	0.87
X	19360309	13933	108	93	18103109	6000	Conserved
X	20521506	8412	172	51	19959934	4824	Conserved
X	20656966	1798	60	22	19001242	286	0.71
X	20656966	1998	60	24	19001242	744	0.59
X	20756081	2631	108	25	18950451	557	0.6
X	20982252	1793	112	16	19277140	433	Conserved
X	21765152	611	122	5	19959934	9404	Conserved
X	22074000	630	187	3	20196957	636	0.73
X	22076628	216	29	4	20199584	210	0.78
X	22094022	206	30	3	20218604	120	Conserved
X	22095960	166	33	5	20220852	232	Conserved
X	23577050	528	132	4	21287252	641	Conserved
